# Integrative metabolomic and transcriptomic analysis reveals stage-specific shifts in hepatic lipid metabolism of broiler chickens

**DOI:** 10.1080/10495398.2026.2622124

**Published:** 2026-02-03

**Authors:** Jingjing Li, Yidan Xu, Peng Ren, Xia Xiong, Mohan Qiu, Chunlin Yu, Yiping Liu, Chaowu Yang

**Affiliations:** a College of Life Science and Agri-forestry, Southwest University of Science and Technology, Mianyang, Sichuan, China; b Animal Breeding and Genetics Key Laboratory of Sichuan Province, Sichuan Animal Science Academy, Chengdu, Sichuan, China; c Farm Animal Genetic Resources Exploration and Innovation Key Laboratory of Sichuan Province, Sichuan Agricultural University, Chengdu, Sichuan, China

**Keywords:** Age, lipid metabolite, metabolic pathways, gene expression, integration analysis

## Abstract

Managing fat accumulation is a critical goal in the poultry industry, with the liver being the primary site for lipid metabolism in chickens. This study used non-targeted metabolomics to investigate dynamic changes in metabolite composition in chicken liver across five physiological stages. A total of 1121 metabolites were identified, with 749 and 372 detected in positive- and negative-ion modes, respectively. The regulation of hepatic lipolysis involves numerous metabolic pathways, making it a complex process. We performed trend analysis of lipid, carbohydrate and amino acid-related metabolites. Age exerted major effects on hepatic metabolism, significantly enriching pathways including alpha-linolenic acid metabolism, linoleic acid metabolism, steroid hormone biosynthesis, 2-oxocarboxylic acid metabolism and nicotinate and nicotinamide metabolism. Transcriptomic analysis identified 12 lipid-metabolism-related genes, showing a tendency of continuously increase. Potential functional genes influencing lipid metabolism–related pathways were identified through a comprehensive analysis of differential lipid-related metabolites and genes, including *fatty acid desaturase 2* (*FADS2*), *acetyl-CoA acetyltransferase 2* (*ACAT2*), *apovitellenin 1* (*APOV1*), *vitellogenin 1* (*VTG1*), *membrane-bound O-acyltransferase domain-containing protein 2* (*MBOAT2*) *and ELOVL fatty acid elongase 1* (*ELOVL1*). These findings could help improve understanding of hepatic metabolism during different physiological stages and identify valuable biomarkers for specific metabolite accumulation.

## Introduction

In modern chicken breeding schemes, fat deposition is a crucial aspect.^1^ There is always an increase in body fat as the chicken grows.^2^ Excessive accumulation of abdominal fat causes a negative impact on consumer acceptance and health.^3^ However, intramuscular fat has been reported to strongly improve meat flavour, juiciness and tenderness.^4^ Therefore, regulating fat deposition is still a main objective in poultry production to adapt to market needs. Lipid accumulation is the result of lipogenesis and lipolysis with complex molecular mechanisms.^5^ In contrast to mammals, lipogenesis in the adipose tissue of chickens is very limited.^6^ The liver is the main site for over 70% of chicken *de novo* fatty acid synthesis.^7^ Fatty acids produced in the liver are incorporated into triacylglycerols and then released as very low-density lipoprotein (VLDL). Then, VLDL travels through the bloodstream to be stored and utilized in muscle and adipose tissue.^8^ Thus, fat deposition in chicken is closely related to hepatic lipid metabolism. Gaining a deeper understanding of hepatic lipid metabolism and elucidating the fundamental mechanisms regulating fat deposition in poultry holds great importance.

Metabolomics is defined as the comprehensive quantitative and qualitative analysis of small-molecule metabolites in biological systems.^9^ It can reflect the down-stream of genes and proteins by measuring the metabolites present in a given biological sample.^10^ RNA sequencing (RNA-Seq) detects gene mRNA expression profiles in different tissues or at different stages of development. Genes regulate the synthesis and accumulation of metabolites.^11^ Some studies have identified specific metabolites and candidate genes associated with hepatic lipid metabolism in chickens under certain conditions or stages.^12–14^ For example, Tang et al. identified glyceric acid, palmitic acid, cis-9-palmitoleic acid and linolenic acid as key metabolites in relation to breed differences.^12^ Xu et al. revealed the role of *EHHADH* in reducing hepatic lipid deposition during fasting in chicken.^13^ Li et al. reported that the *NADB-LER* genes were involved in hepatic lipid metabolism in hens in the peak-laying stage.^14^ However, little is known about the gene-to-metabolite correlations at different physiological stages. In this study, we employed a combined approach of non-targeted metabolomics and RNA-Seq technology to investigate the dynamic changes of metabolites and gene expression profiles in chicken liver during different growing and developing periods. By integrating metabolomic and transcriptomic analyses, we explored the dynamic changes of hepatic lipid metabolism during different physiological stages, including market age, body maturity and sex maturity. We selected the liver sampling ages of 60, 90, 120, 150 and 180 days. Through this integrative approach, we searched for genes significantly correlated with key metabolites. The age-dependent variations in gene expression and metabolites will provide information that may contribute to a better comprehension of the molecular regulatory mechanism of chicken hepatic lipid metabolism.

## Materials and methods

### Animals and liver tissue samples preparation

A total of 200 female Daheng broilers were obtained from Sichuan Da-Heng Poultry Breeding Company (Chengdu, China). All chickens were caged and kept in the same environment with free access to food and water throughout the period. Nine chickens from each group were selected at random and slaughtered for the collection of samples at 60, 90, 120, 150 and 180 days of age. Further, the right lobe of liver was used for histological characteristic analysis. The left lobe of liver tissue within the same region were dissected, snap-frozen in liquid nitrogen and stored at −80 °C until further analysis.

### Histological characteristics of chicken liver development

The liver tissues were immersed in a 4% paraformaldehyde solution (Beyotime) for 24h. After staining with haematoxylin and eosin (Sigma-Aldrich), the tissue sections underwent histological and morphological examination. Oil red O (Sigma-Aldrich) was used to stain frozen sections of liver tissue for 10 minutes. Microscopic images were taken with a digital microscopy (BA400Digital, China) fitted with an image analyser (Image-Pro Plus 6.0).

### Untargeted metabolomics study based on liquid chromatography tandem mass spectrometry (LC-MS/MS)

The liver tissues (100 mg) were grounded with liquid nitrogen and the mixture was resuspended in precooled 80% methanol and 0.1% formic acid. Incubation for 5 min followed by centrifugation at 15000 *g* for 20 min at 4 °C. Supernatants were diluted with LC-MS grade water to a final concentration of 53% methanol and then were centrifuged at 15000 *g* for 20 min at 4 °C. Finally, the supernatants were analysed by LC-MS/MS. The untargeted metabolomics was carried out by Novogene Genetics Technologies Company (Beijing, China) as described previously.^15^ Briefly, an UHPLC system with a Thermo Accucore C30 column (150 × 2.1 mm, 2.6 µm) coupled to an Orbitrap Q Exactive^™^ high-field (HF) mass spectrometer (Thermo Fisher, Germany) was used for lipidomics analysis. 0.1% formic acid (A) and methanol (B) were used as mobile phases for the positive polarity mode. The negative polarity mobile phase were 5 mmol/L ammonium acetate (A) and methanol (B). ExactiveTM HF-X mass spectrometer was operated in positive/negative polarity mode with spray voltage of 3.2 kV, sheath gas flow rate of 40 arb, aux gas flow rate of 10 arb and capillary temperature of 320 °C.

Compound Discoverer 3.1 was used to process the raw data file. Peak comparison, peak picking and quantification of each metabolite were performed. Peak intensities were standardized to the total spectral intensity. The identification of compounds was first predicted from additive ions, molecular ion peaks and fragment ions using the normalized data. The peaks were then checked against the mzCloud, mzVault and MassList databases. This provided relative quantitative and accurate qualitative results. Statistical analyses were performed using R software version 3.4.3. The processed data were imported into MetaX software. Principal components analysis (PCA) and orthonormal partial least squares discriminant analysis (OPLS-DA) were performed. For each multivariate model, the calculated R2 value indicates goodness of fit. The OPLS-DA parameter Q2 represents the predictive ability of the model.^16^ The fold change (FC) value of each metabolite was calculated by comparing the mean value between every two groups. The metabolites with variable importance in projection (VIP) of the first principal component of the OPLS-DA model exceeding 1, FC ≥ 1.5 or FC ≤ 0.667, and *P* value <0.05 were set as differential metabolites. Z-score (standard score) is a value converted based on the relative content of metabolites, which is used to measure the relative content of metabolites on the same level. The z-score plot was used to calculate the relative abundance of metabolites for trend analysis among five age stages. The functions of these metabolites were studied with the aid of the KEGG database.

### RNA extraction, transcriptome analysis and quantitative real-time PCR (qRT-PCR)

Total RNA was isolated from the liver tissues using Trizol reagent (ThermoFisher, USA) according to the manufacturer’s instructions. Agilent 2100 Bioanalyzer (Agilent Technologies) was conducted to validate the integrity of RNA. The purity of RNA was determined by the NanoPhotometer spectrophotometer (ThermoFisher). After RNA integrity and purity were determined, qualified samples were considered acceptable for RNA-Seq. The raw data were cleaned by removing sequence adapters and low-quality reads. FastQC v0.10.1 was run for raw data quality assessment. Reads were then mapped to the GRCg6a chicken reference genome using HISAT2 v2.2.4. DESeq2 package (v 1.24.0) was adopted to analyse the differential expression of transcripts. Genes were considered differentially expressed genes (DEGs) with a false discovery rate (FDR) value < 0.05. Temporal expression profiles among five age stages were conducted using Short Time-series Expression Minern (STEM) (Carnegie Mellon University, USA).

We randomly selected eight DEGs for qRT-PCR verification in each age comparison, and the expression profiles of representative genes among five age stages were also selected for qRT-PCR analysis. The primers used in this study were listed in Table S1. The qRT-PCR was conducted in a CFX-96 (Bio-Rad, Inc., Richmond, CA, USA) qRT-PCR system with a 10 µL reaction volume (1 µL of cDNA, 0.5 µL of the forward and reverse primers (10 µM), 5 µL of TB Green Premix Ex Taq (Tli RNase H Plus) (TaKaRa) and 3 µL of double-distilled H_2_O). All mRNA expression levels were normalized to GAPDH mRNA level. The relative expressions were calculated by the 2^−ΔΔCt^ method, and three biological replicates were performed on each sample.

### Transcriptome, metabolome and statistical analysis

A one-way ANOVA was used to assess differences in gene and metabolite levels among the five groups by using SPSS (v 26.0). All results are expressed as mean ± standard error (SE). Statistical significance was defined as a *P*-value less than 0.05. GO functional categories associated with lipid-metabolism related genes were identified using the GO database (http://www.geneo ntology.org/). Cytoscape (v3.7.0) was utilized to build the network diagram relationship between DEGs and their associated GO functional classes. Pairwise Pearson’s correlation coefficients between lipid-metabolism-related genes and metabolites were calculated by R version 3.5.1. The R package complex_heat_map was used to visualize the correlation coefficients using heatmap plots.

## Results

### Histological changes of chicken liver development

**Figure 1.**
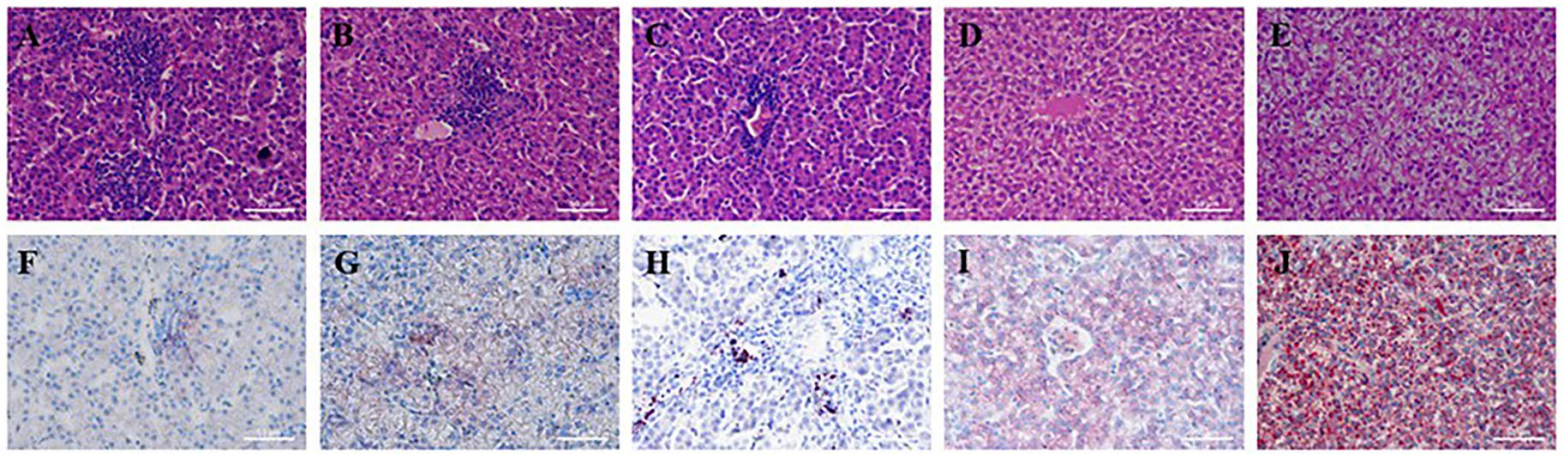
Histological evaluation and oil red O staining of chicken liver among five age stages. (A-E) Histological evaluation of chicken liver with day 60, day 90, day 120, day 150 and day 180 (400×). (F-J) Oil red O staining of chicken liver with day 60, day 90, day 120, day 150 and day 180 (400×). the red part after oil red staining is the lipid droplets.

As shown in Figure 1(A–J), the hepatocytes were arranged neatly and clearly. The nucleus of the hepatocytes was blue, and the cytoplasm was pink. With the increase in the chicken age, the hepatocytes were gradually enlarged. At 180 days of age, we observed that the cytoplasm got loosened and lightly stained. Histochemical analysis using oil red O staining revealed a clear age-dependent progression of lipid accumulation. At 60 days of age, little lipid droplets were stained with oil red O. The lipid droplets were gradually growing and increasing. The numerous small lipid droplets appeared in the liver at day 150, and their sizes were rapidly increased at day 180. This histological evidence of escalating lipid deposition lays a foundation for our subsequent metabolomic investigation into the underlying molecular changes

### Differential metabolites identification

**Figure 2.**
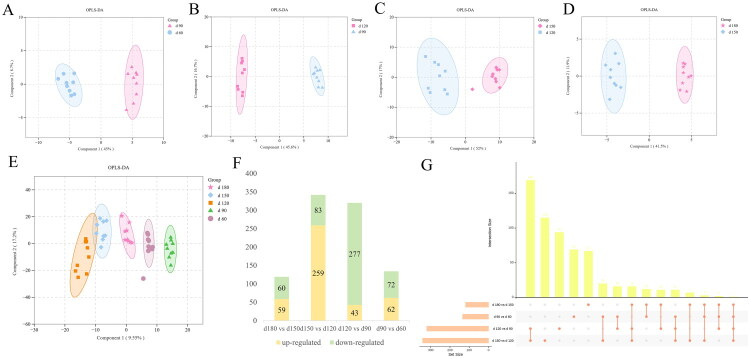
Metabolomics analysis of liver from female daheng broilers of different ages. (A-D) The OPLS-DA revealed significant differential metabolites between two adjacent age groups (d 60 vs d 90; d 90 vs d 120; d 120 vs d 150; d 150 vs d 180). (E) OPLS-DA scores of chicken liver metabolites among five age groups. (F) The numbers of differential metabolites in different comparison groups. (G) Upset venn diagram of differential metabolites in different comparison groups.

The OPLS-DA model showed a clear distinction between two adjacent age groups (Figure 2(A–D)). The OPLS-DA model showed a clear distinction among five age groups (Figure 2(E)). The values of R2Y and Q2Y demonstrated OPLS-DA models had the good cumulative ability to interpret and predict. Permutation testing was used to validate the quality of the model (Figure S1(A–E)).

The VIP values from the OPLS-DA model comparing the five age groups were used to screen for differentially abundant metabolites with biological significance. Based on VIP > 1 and *P* value < 0.05 and FC ≥ 1.5, or FC ≤ 0.667, a total of 749 and 372 metabolites with qualitative names were identified in the positive and negative modes, respectively. In this study, we combined the metabolomics data in positive and negative mode for later analysis. A total of 62(72), 43(277), 259(83), 59(60) metabolites were significant upregulated (downregulated) in the comparison of d 90 vs d 60, d 120 vs d 90, d 150 vs d 120, d 180 vs d 150, respectively (Figure 2(F)). Consistent with the marked histological changes observed between days 120 and 150, the livers of chicken from day 120 to day 150 undergone the greatest metabolic alterations, as indicated by the highest numbers of differential metabolites (Figure 2(G)). Glyceraldehyde 3-phosphate diethyl acetal was identified as the only shared differential metabolite among four age group comparisons. Interestingly, we found 169 shared differential metabolites identified in day 120 *vs* day 90 and day 150 *vs* day 120 comparisons. Of these, 25 metabolites were lipid and derivatives. Considering the important role of chicken liver in fatty acids synthesis, differential metabolites annotated to ‘lipids and lipid-like molecules’ database are shown in the Table S2.

### Analysis of key metabolites

Based on the meta-intensity of metabolomic data across five age groups, the results of the clusters revealed 6 patterns of variation. Cluster 5 exhibited a general increase as age progressed (Figure S2), and a total of 19 metabolites were enriched into cluster 5. Here, Table 1 lists the metabolites screened from cluster 5. Among the five age groups, there were 19 metabolites increased with the increase in slaughter age. Conversely, there were 18 metabolites decreased with the decrease in slaughter age. Types of these metabolites included fatty acyls, glycerophospholipids, sphingolipids, sterol lipids.

**Table 1.** Metabolites from clusters 5 and 6 were annotated in the lipid maps database.

Compounds ID	Compounds name	Formula	Category	RT [min]	*m*/*z*
**increased with slaughter age among five age groups**					
Com_3719_neg	Thromboxane B1	C_20_H_36_O_6_	Fatty Acyls	11.7	353.233
Com_6413_pos	1,4-dihydroxyheptadec-16-en-2-yl acetate	C_19_H_36_O_4_	Fatty Acyls	13.4	311.258
Com_110556_pos	Nonadecanoic acid	C_19_H_38_O_2_	Fatty Acyls	0.5	299.294
Com_137074_pos	Pentadecanoic acid	C_15_H_30_O_2_	Fatty Acyls	0.0	243.232
Com_2449_pos	13-HPODE	C_18_H_32_O_4_	Fatty Acyls	13.2	313.237
Com_36_neg	Docosapentaenoic acid	C_22_H_34_O_2_	Fatty Acyls	14.6	329.248
Com_49_pos	Propionylcarnitine	C_10_H_19_NO_4_	Fatty Acyls	3.3	218.139
Com_5968_neg	13,14-dihydro-15-keto-PGD2	C_20_H_32_O_5_	Fatty Acyls	11.7	351.217
Com_796_pos	Docosahexaenoic acid	C_22_H_32_O_2_	Fatty Acyls	15.1	329.247
Com_1048_neg	LPI 22:6	C_31_H_49_O_12_P	Glycerophospholipids	13.9	643.289
Com_110_neg	LPI 20:4	C_29_H_49_O_12_P	Glycerophospholipids	13.9	619.289
Com_366_neg	LPE 22:6	C_27_H_44_NO_7_P	Glycerophospholipids	14.5	524.278
Com_470_neg	LPS 22:6	C_28_H_44_NO_9_P	Glycerophospholipids	13.9	568.268
Com_527_pos	LPC 22:6	C_30_H_50_NO_7_P	Glycerophospholipids	14.5	568.340
Com_5756_neg	LPI 22:4	C_31_H_53_O_12_P	Glycerophospholipids	14.1	647.311
Com_6772_pos	LPE 22:5	C_27_H_46_NO_7_P	Glycerophospholipids	14.7	528.309
Com_4660_pos	SM (d18:0/16:0)	C_39_H_81_N_2_O_6_P	Sphingolipids	15.1	705.591
Com_1447_neg	Glycoursodeoxycholic acid	C_26_H_43_NO_5_	Sterol Lipids	12.8	448.306
Com_1942_neg	Deoxycholic acid	C_24_H_40_O_4_	Sterol Lipids	12.2	391.285
**decreased with slaughter age among five age groups**					
Com_1647_pos	Oleamide	C_18_H_35_NO	Fatty Acyls	14.1	300.289
Com_45220_pos	1,2-dihydroxyheptadec-16-yn-4-yl acetate	C_19_H_34_O_4_	Fatty Acyls	13.5	349.235
Com_42586_pos	Avocadyne 1-acetate	C_19_H_34_O_4_	Fatty Acyls	14.6	349.235
Com_77094_pos	Thromboxane B2	C_20_H_34_O_6_	Fatty Acyls	6.1	393.220
Com_6205_pos	2-Arachidonoyl glycerol	C_23_H_38_O_4_	Glycerolipids	14.5	361.274
Com_5162_neg	LPG 18:3	C_24_H_43_O_9_P	Glycerophospholipids	13.7	505.257
Com_3652_neg	LPG 20:2	C_26_H_49_O_9_P	Glycerophospholipids	14.3	535.304
Com_4672_neg	PG (18:2/18:2)	C_42_H_75_O_10_P	Glycerophospholipids	15.6	769.504
Com_266_neg	LPS 18:2	C_24_H_44_NO_9_P	Glycerophospholipids	13.9	520.268
Com_1050_neg	LPE 20:3	C_25_H_46_NO_7_P	Glycerophospholipids	14.7	502.294
Com_1056_neg	LPE 18:3	C_23_H_42_NO_7_P	Glycerophospholipids	14.4	474.262
Com_2494_neg	LPC 20:2	C_28_H_54_NO_7_P	Glycerophospholipids	15.1	606.378
Com_4519_neg	Xanthohumol	C_21_H_22_O_5_	Polyketides	14.3	353.142
Com_64153_pos	Chrysin	C_15_H_10_O_4_	Polyketides	8.4	255.065
Com_65233_pos	Daidzin	C_21_H_20_O_9_	Polyketides	8.5	417.118
Com_41701_pos	Dehydrocholic acid	C_24_H_34_O_5_	Sterol Lipids	13.8	403.245
Com_3875_pos	Ursodeoxycholic acid	C_24_H_40_O_4_	Sterol Lipids	13.9	393.299
Com_578_neg	Cholic acid	C_24_H_40_O_5_	Sterol Lipids	12.6	407.280

**Figure 3.**
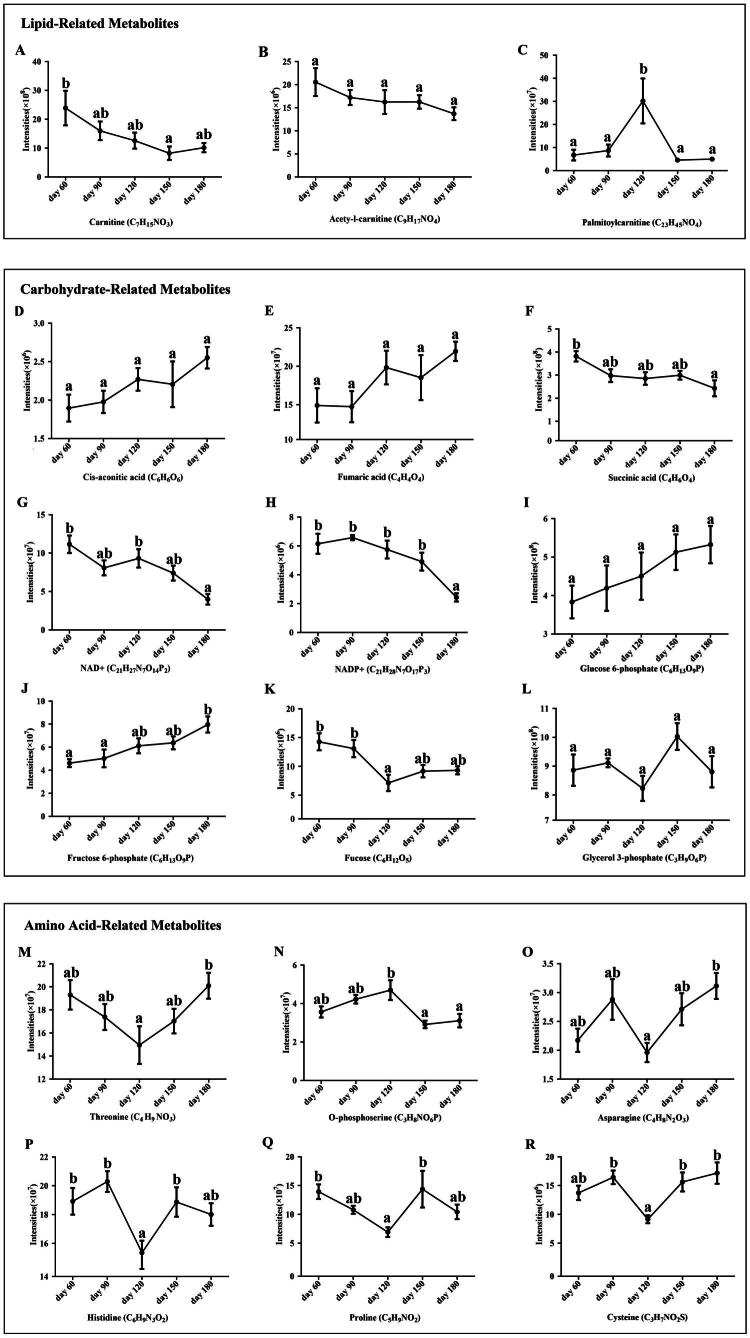
The variation trend of important metabolites among five age stages. The X-axis represents the five age groups, and the Y-axis represents the metabolite intensities. (A-C) Lipid-related metabolites: Carnitine (C_4_H_15_NO_3_), acetyl-l-carnitine (C_9_H_17_NO_4_) and palmitoylcarnitine (C_23_H_45_NO_4_). (D-L) carbohydrate-related metabolites: Fucose (C_6_H_12_O_6_), glycerol 3-phosphate (C_3_H_9_O_6_P), NAD+ (C_21_H_27_N_7_O_14_P_2_), NADP+ (C_21_H_28_N_7_O_17_P_3_), fructose 6-phosphate (C_6_H_13_O_9_P), glucose 6-phosphate (C_6_H_13_O_9_P), succinic acid (C_4_H_6_O_4_). (M-R) amino acid-related metabolites: Threonine (C_4_H_9_NO), histidine (C_6_H_9_N_3_O_2_), proline (C_5_H_9_NO₂), asparagine (C_4_H_8_N₂O_3_), cysteine (C_3_H_7_NO_2_S), O-phosphoserine (C_3_H_8_NO_6_P). Different superscript lowercase letters indicate a significant difference at *P* < 0.05.

Here, we listed the variation trend of important metabolites among five age stages in Figure 3. For lipid-related compounds, the livers of aged chicken had lower content of carnitine and acetyl-l-carnitine than that of young chicken. The palmitoylcarnitine showed the tendency of first increase and then decrease, with the highest content in livers of 120-day-old chickens (*P* < 0.05) (Figure 3(A–C)). For carbohydrate-related metabolites, we observed that two intermediates in glycolysis/gluconeogenesis, including fucose and glycerol 3-phosphate, also showed the lowest concentrations at day 120. However, the levels of nicotinamide adenine dinucleotide (NAD+) and nicotinamide adenine dinucleotide phosphate (NADP+) were significantly decreased from day 120 to day 180 (*P* < 0.05). Fructose 6-phosphate and glucose 6-phosphate presented a total upward trend among five age groups. Succinic acid, an intermediate involved in the tricarboxylic acid cycle (TCA), was found to decrease from day 60 to day 180 (*P* < 0.05) (Figure 3(D–L)). Most amino acids presented a similar trend among five age stages (Figure 3(M–R)). The abundance of threonine, asparagine, histidine and proline, and cysteine were all decreased at day 120, and then sharply increased to day 180 (*P* < 0.05). O-phosphoserine intensity showed a opposite trend: rising from day 60-120, then significant falling through day 150 (*P* < 0.05).

### KEGG pathway enrichment analysis of differential metabolites

**Figure 4.**
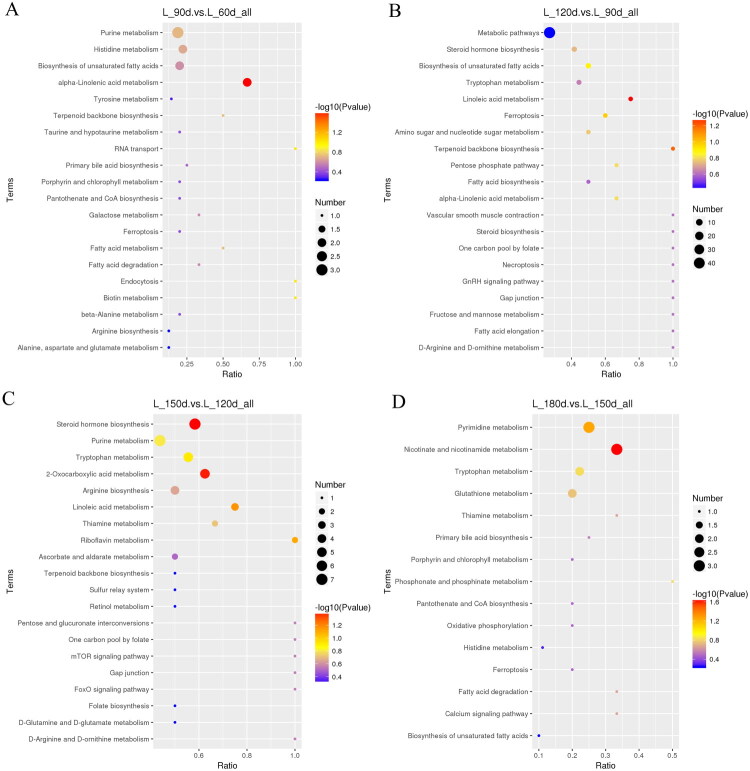
The specific KEGG pathways in which the distinguished metabolites are involved differ across various age groups. (A) day 90 vs day 60; (B) day 120 vs day 90; (C) day 150 vs day 120; (D) day 180 vs day 150. Bubble size represents the number of metabolites enriched in the pathway. The bubble colour represents the degree of significance, from the highest (red) to the lowest (purple).

The differential metabolites were elucidated through the KEGG pathways. Here, the top 20 pathways were displayed of four age group comparisons (Figure 4). In each age comparison, we only focus on significant pathways (*P* < 0.05). In the first stage (day 60–90 day), the alpha-linolenic acid pathway was the most enriched for differential metabolites (Figure 4(A)). From day 90 to day 120, linoleic acid metabolism was the identified pathway (Figure 4(B)). Steroid hormone biosynthesis and in the comparison of gene expression between the 150th day and the 120th day, 2-oxocarboxylic acid metabolism was identified as a significant pathway (Figure 4(C)). Nicotinate and nicotinamide metabolism was the most significant pathway when comparing day 180 and day 150 (Figure 4(D)).

### Differential expressed genes identification and qRT-PCR verification

**Figure 5.**
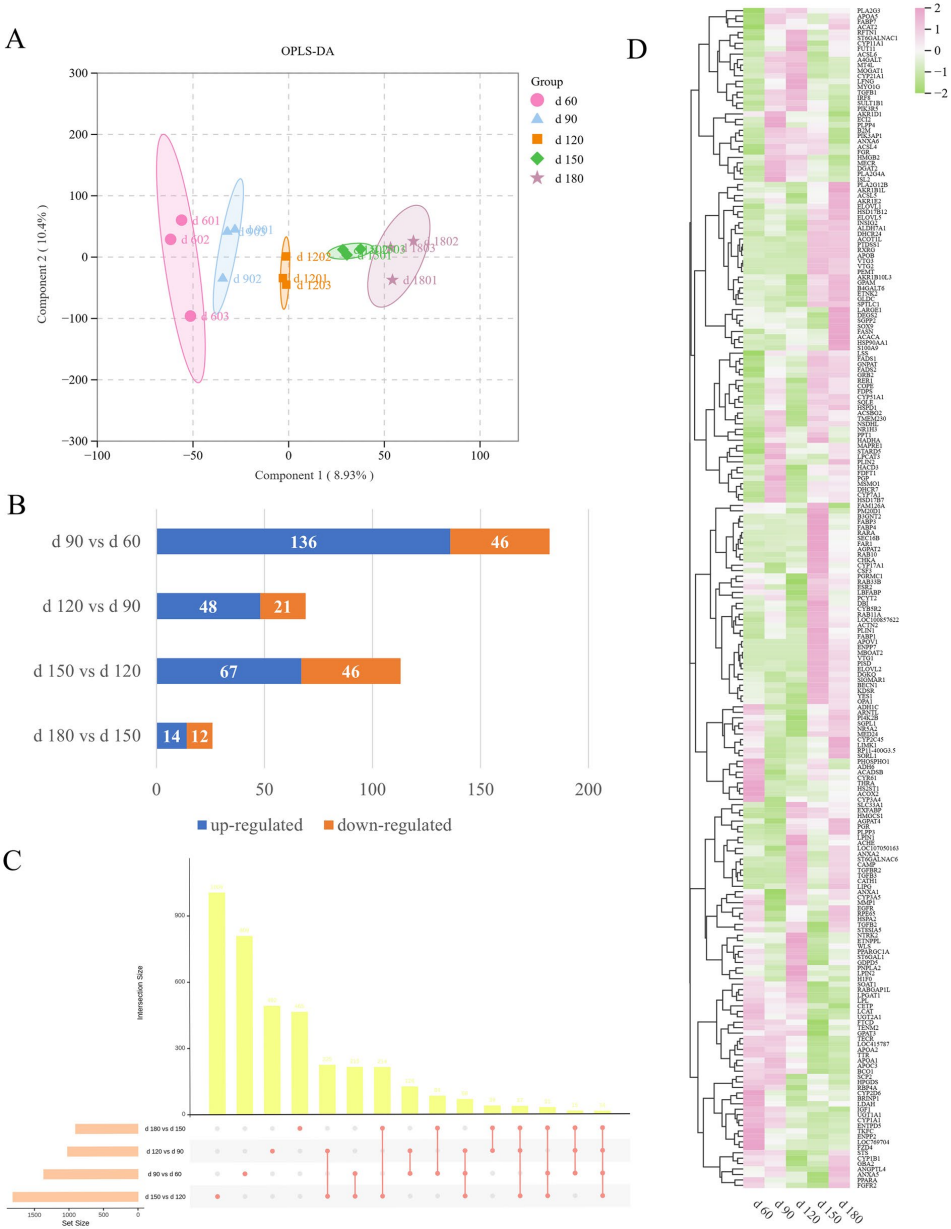
Transcriptome analyses of liver from female Daheng broilers of different ages. (A) OPLS-DA scores of chicken liver genes at 60, 90, 120, 150 and 180 days of age. (B) The numbers of DEGs in different comparison groups. (C) Upset Venn diagram of DEGs in different comparison groups. (D) Heatmap of DEGs for 5 age groups.

We conducted the same age comparisons as that in LC-MS/MS analysis. The OPLS-DA analysis demonstrated discernible principal components among five age groups. PC1 elucidated 8.93% of the observed variability, whereas PC2 contributed to 10.4% (Figure 5(A)). A total of 182, 69, 113 and 26 DEGs were identified, respectively (Figure 5(B)). Between d 180 and d 150, there were the fewest DEGs, while the highest number was observed between d 90 and d 60. Upset Venn graph showed that 14 common DEGs identified in four age comparisons, including *solute carrier family 4 member 4 (SLC4A4), H1.010 linker histone, cluster member (HIST1H110), multiple EGF-like-domains 9 (MEGF9), ATP binding cassette subfamily A member 12 (ABCA12), polypeptide N-acetylgalactosaminyltransferase 16 (GALNT16), fatty acid binding protein 1 (FABP1), protocadherin 1 (PCDH1), tetratricopeptide repeat domain 32 (TTC32), nebulette (NEBL), gamma-aminobutyric acid type A receptor alpha3 subunit (GABRA3), ubiquinol-cytochrome c reductase (BCS1L), complex III subunit XI (UQCR11), dual specificity phosphatase 16 (DUSP16), LOC101747860* and *LOC768772*, of which *FABP1* were related to lipid metabolism (Figure 5(C)). The heatmap in Figure 5(D) showed the DEGs related to lipid metabolism.

**Figure 6.**
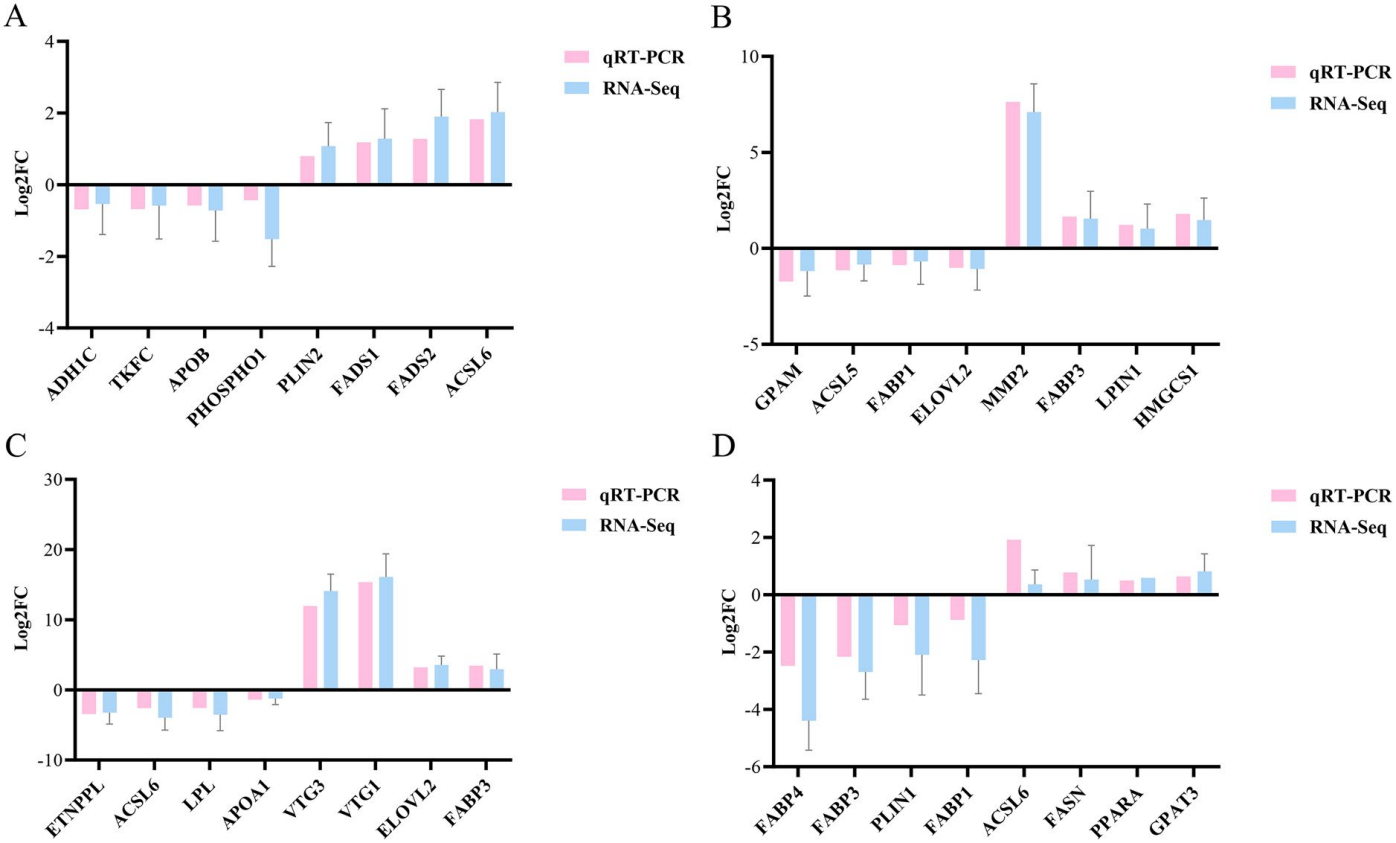
Comparison of the log 2-fold change (FC) for lipid-metabolism related genes between the results of qRT-PCR and mRNA-Seq. (A) day90 vs day60; (B) day120 vs day90; (C) day150 vs day120; (D) day180 vs day150. The X-axis represents different genes. Y-axis represents log 2-fold change. Results are expressed as mean ± standard deviation (*n* = 3).

Eight lipid-metabolism-related genes were randomly selected for validation of the RNA-seq results using qRT-PCR in each age comparison. The results were consistent with the RNA-seq data (Figure 6(A–D)).

### mRNA expressions of lipid-metabolism-related genes

**Figure 7.**
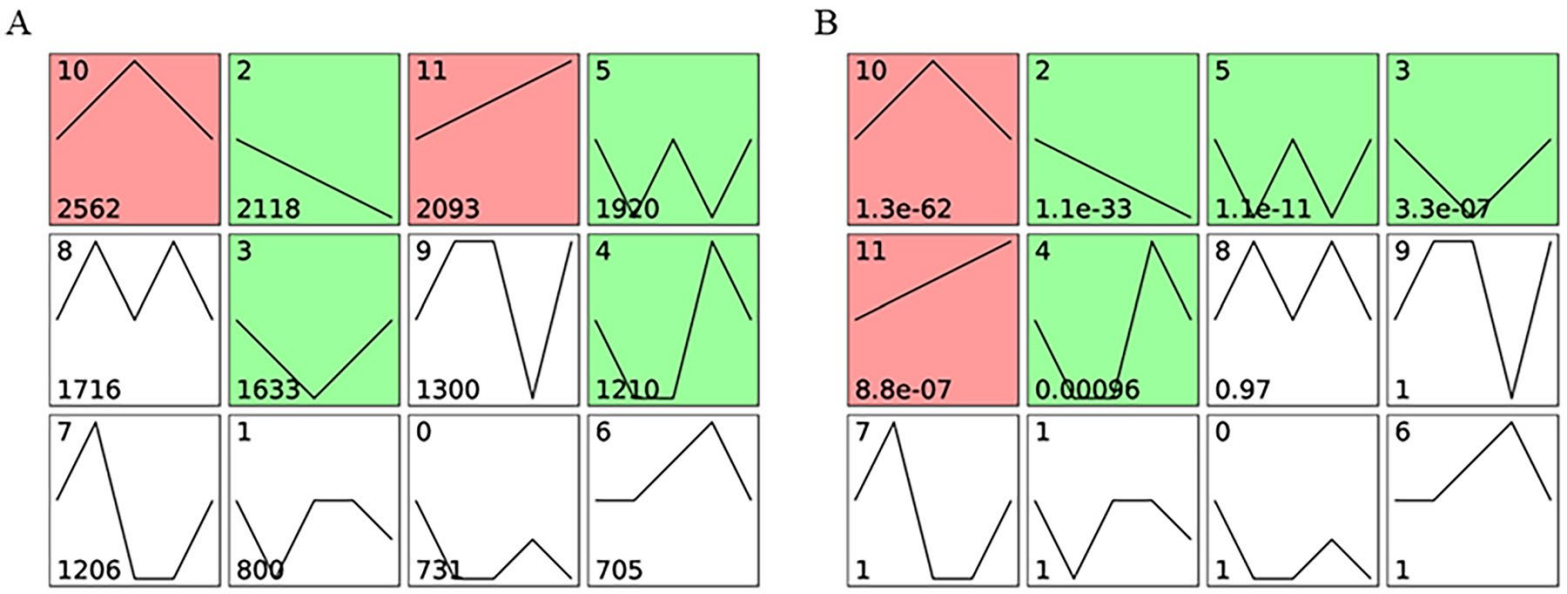
STEM software identified 12 temporal gene expression patterns (0-11), with counts of MEs on the upper left. (A) The bottom number signifies the total count of genes that fall into these temporal expression patterns. (B) The bottom number indicates the statistical significance of the temporal expression patterns. Patterns with significant changes are highlighted in green for upward trends, red for downward trends and white for insignificant trends.

**Figure 8.**
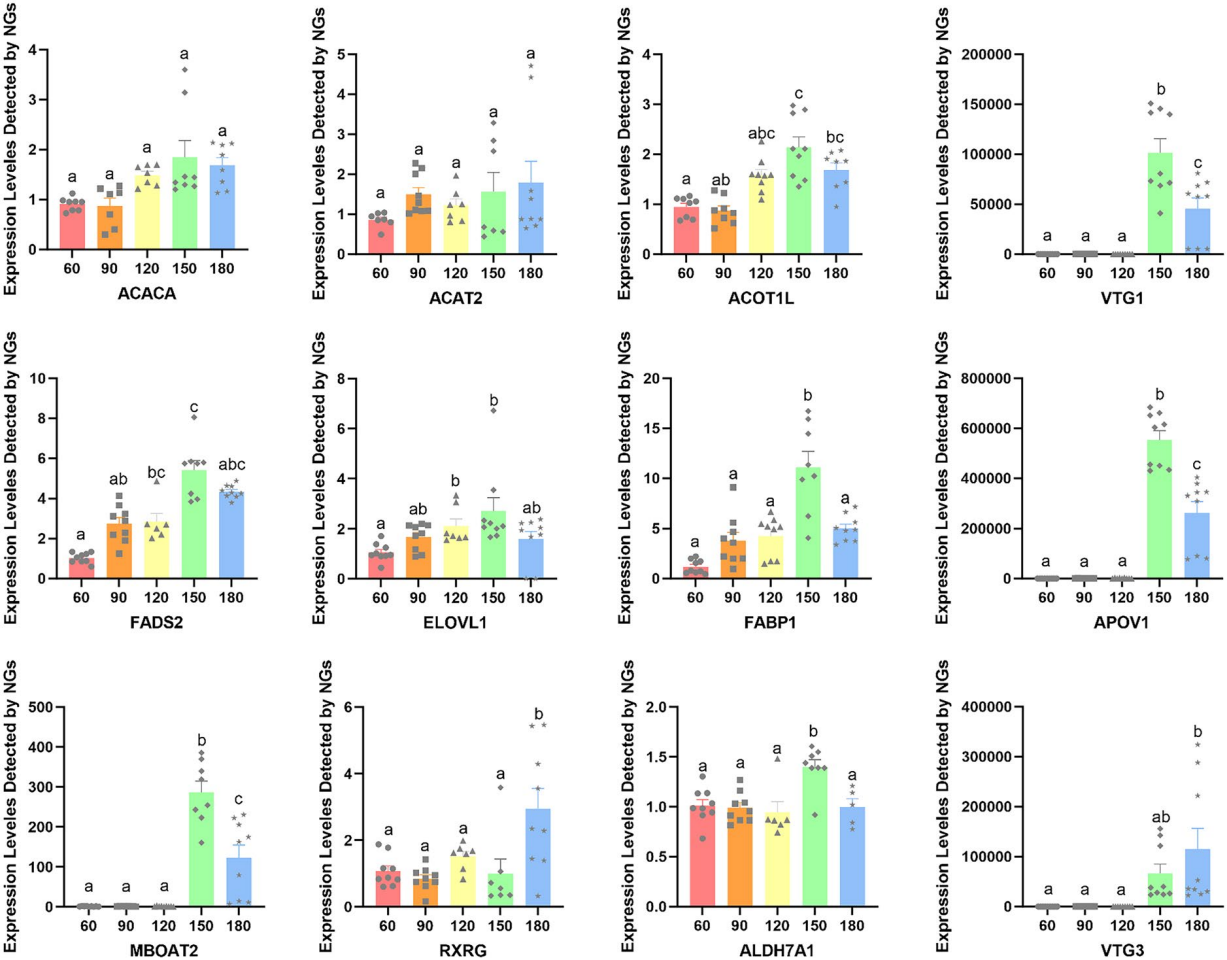
mRNA expressions of 12 lipid-metabolism-related genes enriched into pattern 11. Different superscript lowercase letters indicate a significant difference at *P* < 0.05.

The filtered RNA-seq data were summarized using STEM software to cluster it was categorized into 12 unique temporal expression patterns across five age stages (Figure 7). The bottom number in Figure 7(A) signifies the total count of genes that fall into these temporal expression patterns, while the bottom number in Figure 7(B) indicates the statistical significance of the temporal expression patterns. 6 temporal expression patterns were significantly changed, of which four patterns (2, 3, 4, 5) were found to be downregulated and two patterns (10 and 11) were significantly upregulated (*P* < 0.05). Temporal expression pattern 11 showed a tendency of continuously increase from day 60 to day 180. For the reason that the lipid droplets were gradually growing and increasing, we selected 12 lipid-metabolism-related genes enriched into pattern 11 for qRT-PCR verification. Although these genes showed a tendency of continuously increase, there was no significant change in mRNA expressions of *acetyl-CoA carboxylase alpha (ACACA), acetyl-CoA acetyltransferase 2 (ACAT2), acyl-CoA thioesterase 1 like (ACOT1L), vitellogenin 1 (VTG1), fatty acid binding protein 1 (FABP1), apovitellenin 1 (APOV1), membrane-bound O-acyltransferase domain-containing protein 2 (MBOAT2), retinoid X receptor gamma (RXRG), aldehyde dehydrogenase 7 family member A1 (ALDH7A1)* from day 60 to day 150 (*P* > 0.05). The expressions of most genes, including *VTG1, fatty acid desaturase 2 (FADS2), FABP1, APOV1, MBOAT2,* and *ALDH7A1*, were significantly increased at day 150, but subsequently declined from day 150 to day 180 (*P* < 0.05). Two genes, *retinoid X receptor gamma (RXRG)* and *vitellogenin 3 (VTG3)*, showed the highest expressions at 180 days of age (*P* < 0.05) (Figure 8).

### DEGs-pathway network and correlation analysis between lipid-metabolism-related genes and metabolites

**Figure 9.**
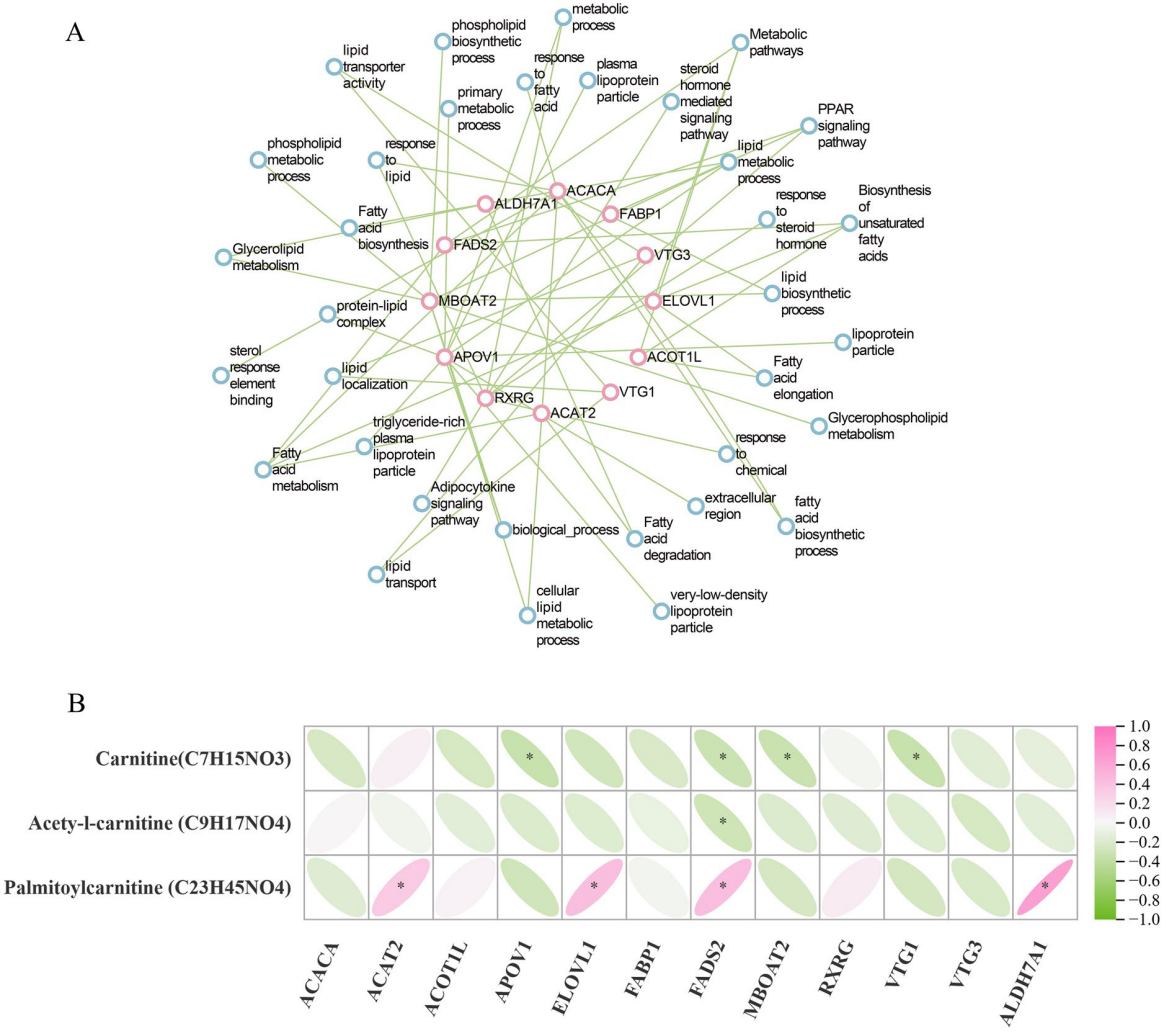
(A) Relationship network diagram between lipid-metabolism-related genes and pathways. The nodes in pink colour represent DEGs. The nodes in blue colour represent lipid-metabolism-related pathways. (B) Correlation analysis between lipid-related metabolites and gene expression levels. *P < 0.05.

Here, we conducted the enrichment of 12 lipid-metabolism-related genes and GO pathway integrative analysis. Figure 9(A) showed that *FABP1, FADS2* and *RXRG* genes were involved in the PPAR signalling pathway. Two genes (*VTG1* and *VTG3*) regulated lipid transporter activity, lipid localization and lipid transport. The lipid metabolic process was controlled by genes such as *MBOAT2* and *APOV1. ACACA* genes regulated the fatty acid biosynthetic process, but *ACAT2* and *ALDH7A1* genes regulated the fatty acid degradation. *ACOT1L* and *ELOV1* gene controlled metabolic pathways (fatty acid elongation and biosynthesis of unsaturated fatty acids). These 12 DEGs are all related to the synthesis and metabolism of lipids.

The correlations between differential metabolites and gene expressions were investigated. Here, we focused on lipid-related metabolites and 12 lipid-metabolism related genes. As shown in Figure 9(B), there was no significant correlation between differential lipids metabolites and *ACACA, ACOT1L, FABP1, VTG3* and *RXRG.* Palmitoylcarnitine was significantly and positively correlated with *ACAT2, ELOVL1, FADS2, ALDH7A1* (*P* < 0.05). Carnitine was significantly and negatively correlated with *APOV1, FADS2, MBOAT2,* and *VTG1* (*P* < 0.05). *FADS2* gene was negatively correlated with acetyl-l-carnitine (*P* < 0.05)

## Discussion

The liver is the pivotal regulator of lipid metabolism in chickens, accounting for over 70% of de novo fatty acid synthesis.^16^^,^^17^ It orchestrates key processes including lipid synthesis, transport and degradation.^17^ The chicken’s liver produces apolipoprotein and vitellogenin, which constitute the primary constituents of the egg yolk.^18^ Previous research has investigated the morphological and histological characteristics of the liver, as well as the differences in expression of genes related to lipid metabolism between juvenile and laying hens.^17^ For chicken breeder, it is significant to investigate liver lipid metabolism across a wider age spectrum from day 60 to day 180 to determine its mode of action of chicken.

Gradual morphological and histological changes occur in the liver during the growth and development process. The large number of small lipid droplets appeared in the liver at day 150, which indicated that the lipogenesis increased after sexual maturity. Cui et al. suggested that the key period for hepatic lipid synthesis was from day 139 to day 153.^19^ During sexual maturity period, we found that hepatic lipid synthesis dramatically increased from day 120 to day 150. After sexual maturity, hepatic lipid synthesis continued to increase to day 180.

Lipid metabolism encompasses a intricate network of various metabolic routes. Utilizing metabolomics, a holistic systems-based strategy, researchers have gained insights into the dynamics of lipid metabolism during the fat accumulation phase of animal growth.^20–22^ To identify changes in lipid metabolism, a comprehensive analysis of metabolite changes among five age groups was performed using liver metabolic profiling in this study. The metabolites involved in plenty of metabolic pathways including the amino acids metabolism, glycolysis, gluconeogenesis, fatty acid oxidation. The livers of chicken from day 120 to day 150 undergone the greatest changes, with most differential metabolites identified in this stage. As hens reach sexual maturity, liver metabolism may increase to support the requirements for egg yolk production.^17^

Liver mitochondria used free fatty acids in β-oxidation and for ATP production. Carnitine is involved in transferring fatty acids across the mitochondrial membrane for subsequent β-oxidation.^23^ Carlson et al. showed that dietary l-carnitine supplementation increased liver carnitine concentrations and increased in vitro fatty acid β-oxidation in liver slices of cows.^24^ Acetyl-l-carnitine (ALC) is an ester of L-carnitine and helps to uptake of acetyl CoA into the mitochondria during fatty acid oxidation. A previous study also reported that liver mitochondrial content and function were improved in oral feeding of ALC to rodents.^25^ The present study showed that the livers of aged chicken had lower content of carnitine and acetyl-l-carnitine than that of young chicken. Thus, the fatty acid oxidation was weaker in the livers of old chicken. Fatty acid oxidation gradually decreased in chicken livers during growth and development process. In this study, the increased fat accumulation in aged chicken liver was derived from 19 lipid metabolites, which were enriched into cluster 5 and increased with age. These metabolites included fatty acyls, glycerophospholipids, sphingolipids, sterol lipids. The observed increase in lipid metabolites, coupled with a decrease in their metabolic intermediates, points towards an age-dependent accumulation of fat, mediated by a decline in β-oxidation.

Besides the essential role in protein synthesis, amino acids were directly associated with a wide range of biological processes. In this study, the levels of most amino acids, peptides, and analogues metabolites presented fluctuant tendency during growth and development process. Histidine is an essential animo acids for humans. It is reported that histidine has antioxidant functions and plays particularly important roles in the active site of enzymes.^26^^,^^27^ Excess histidine can be catabolized by histidine ammonia lyase (histidase) in liver.^26^ Proline could effectively protect the liver from liver injury. Novak et al. reported that feeding the low-protein diet might have played a role in decreasing egg production.^28^ The increased amino acids content from day 120 was probably due to the need to adapt to the subsequent egg production. However, a sharp decrease in O-phosphoserine content was observed from day 120. O-phosphoserine is a precursor substance to form plentiful amino acids, such as glycine, serine, threonine.^29^^,^^30^ The increase in the level of O-phosphoserine from day 60 to day 120 showed that the broilers in this stage needed to reserve more substances to generate more amino acids for subsequent egg production. The decrease in O-phosphoserine from day 120 explains why most amino acids increased during this stage.

Nicotinamide adenine dinucleotide (NAD+) is a substrate for major dehydrogenase enzymes involved in nutrient catabolism. The phosphorylated form NADP+ is electron provider of power mitochondrial oxidative phosphorylation.^31^^,^^32^ In a study of human across a wide age range, results showed that a significant decline in the plasma levels of NAD+, NADP+ with age.^33^ However, in Figure 3, we find that NAD+ and NADP+ were increased from day 60 to day 120 and then decreased from day 120 to day 180 in chicken liver. The different change trend of NAD+ and NADP+ age may be due to variant nutrient catabolism in mammals and vertebrates. Glucose is a crucial monosaccharide essential for energy production, and glucose metabolism is ubiquitous in all cells.^34^ Intracellular glucose is phosphorylated to glucose-6-phosphate and then transformed into glycogen or metabolized through glycolysis pathway.^35^ The contents of glucose 6-phosphate and fructose 6-phosphate, presented a total upward trend among five age groups. Glucose-6 phosphate is the first intermediate of glucose metabolism and plays a central role in the energy metabolism of the liver.^36^ These findings indicate that amino acid utilization and the effectiveness of glycolysis/gluconeogenesis were adjusted to meet the nutritional requirements of various physiological stages.

In chicken, more than 70% of *de novo* fatty acid synthesis takes place in the liver.^37^ Lipogenic activity in chickens is much greater in the liver than in adipose tissue.^37^ To identify and characterize genes that control fat deposition, transcriptome was conducted in this study. A total of 12 distinct temporal expression patterns were identified among five age stages, of which pattern 11 showed a tendency of continuously increase. For the reason that the lipid droplets were gradually growing and increasing, 12 lipid-metabolism-related genes enriched into pattern 11 were selected for qRT-PCR verification. The *ACACA* gene encodes acetyl-CoA carboxylase alpha, which catalysed the first committed step of fatty acid synthesis, leading to the biosynthesis of long-chain fatty acids.^38^^,^^39^ The balance between lipogenesis and export of VLDL particles in liver is important for egg production.^40^ *APOV1* is major transport proteins of VLDL. Richards et al. proposed that hepatic expression of *APOV1* genes increased significantly in broiler breeders at first egg.^41^ FABPs are known to bind free fatty acids and transport them to different organelles for lipid metabolism or storage.^42^ Wang et al. discovered that the expression levels of *FABP1* underwent a significant increase between the ages of 20 weeks (pre-laying period) and 30 weeks (peak laying period).^43^ Figure 9(A) also shows that these genes collaborate to regulate lipid metabolism and synthesis. Consistent with previous study, mRNA expressions of *ACACA, APOV1* and *FABP1* showed continuous upward trend from 60 days old to 150 days old.

Integrated analysis helps identify potential gene function for specific metabolite accumulation and biological processes. For the subsequent correlation analysis, we concentrated solely on lipid-related metabolites and lipid-metabolism-related DEGs. A key finding was the significant positive correlation between palmitoylcarnitine and the genes *FADS2*, *ACAT2* and *ELOVL1* (*P* < 0.05). This correlation network suggests a coordinated molecular programme favouring lipid synthesis over catabolism.^44–46^ *FADS2* encodes a rate-limiting enzyme involved in fatty acid desaturation and the production of long-chain polyunsaturated fatty acids, such as arachidonic acid.^44^^,^^45^ Its elevated expression is a known feature in human non-alcoholic fatty liver disease (NAFLD), where it drives pro-inflammatory lipid mediator synthesis.^46^^,^^47^ In our study, the co-upregulation of *FADS2* with *ELOVL1* (which elongates fatty acids) and *ACAT2* (which esterifies cholesterol) aligns with a conserved lipogenic response, potentially explaining the observed hepatic lipid droplet expansion.^47^^,^^48^ Thus, palmitoylcarnitine accumulation here may not solely reflect β-oxidation but also feedforward stimulation of lipogenesis during metabolic stress of rapid growth and sexual maturation.^49^

Conversely, we observed a significant negative correlation between carnitine and the genes *APOV1, FADS2, MBOAT*, and *VTG1* (*P* < 0.05). Carnitine is a key cofactor in fatty acid β-oxidation, and its depletion is linked to impaired fat catabolism.^49^ *MBOAT2* encodes an enzyme that esterifies lysophospholipids, and its overexpression promotes hepatic triglyceride synthesis.^50^ The inverse relationship with carnitine suggests a metabolic trade-off wherein lipogenesis is upregulated as fatty acid oxidation is suppressed.^49^^,^^50^ Similarly, *APOV1* facilitates lipoprotein assembly, and its negative correlation with carnitine aligns with studies linking dyslipidaemia (e.g. elevated LDL-C) to reduced carnitine availability in metabolic dysfunction-associated steatotic liver disease.^47^ Besides, *VTG1,* a yolk precursor protein involved in lipid transport, also showed the same inverse association.^51^ Notably, *FADS2* was negatively associated with both carnitine and acetyl L-carnitine, suggesting that it may affect lipid metabolism through a dual mechanism: on the one hand, it promotes polyunsaturated fatty acid synthesis, and on the other hand, it inhibits carnitine-mediated fatty acid oxidation, leading to an imbalance in lipid metabolism.^44^^,^^45^ Collectively, these findings position *FADS2* as a critical node in lipid metabolic dysregulation, where its overexpression may drive hepatic lipogenesis and inhibit fatty acid oxidation.

While this study provides novel insights into the age-dependent dynamics of hepatic lipid metabolism in broilers, certain limitations should be acknowledged. Firstly, the conclusions drawn are based exclusively on female broilers. Therefore, the generalizability of our findings to male chickens or other poultry breeds requires further investigation. Secondly, although our sampling time points (60, 90, 120, 150 and 180 days) covered crucial stages from growth to sexual maturity, the key metabolic shifts appeared concentrated between 120 and 150 days of age. Future studies employing a denser sampling frequency during this critical window would help delineate the dynamic details and regulatory mechanism of metabolic transitions.

## Conclusion

In summary, the liver lipogenesis increased with age, especially after sexual maturity. Additionally, these altered metabolites were involved in lipid, amino acid and energy metabolism. The primary metabolic pathways affected by age in chicken liver were alpha-linolenic acid metabolism, linoleic acid metabolism, steroid hormone biosynthesis and nicotinate and nicotinamide metabolism. Gene trend analysis and qRT-PCR verification showed a continuous upward trend in the mRNA expression of several lipid metabolism-related genes. Most importantly, integrated analysis of lipid metabolism-related metabolites and genes identified a set of potential functional genes—including *FADS2*, *ACAT2*, *APOV1*, *VTG1*, *MBOAT2* and *ELOVL1*—that appear to coordinately regulate hepatic lipid metabolism to meet the physiological demands at different stages.

## Supplementary Material

Original Image for Fig 1G.jpeg

Original Image for Fig 1C.jpeg

Original Image for Fig 1I.jpeg

Original Image for Fig 1H.jpeg

Original Image for Fig 1B.jpeg

Original Image for Fig 1E.jpeg

Original Image for Fig 1F.jpeg

suplementary materials.docx

Original Image for Fig 1D.jpeg

Original Image for Fig1A.tif

Original Image for Fig 1J.jpeg

## Data Availability

Sequence data that support the findings of this study have been deposited in the NCBI Sequencing Read Archive (SRA) under Biological Programs PRJNA1134808.

## References

[1] Cui H, Liu R, Zhao G, et al. Identification of differentially expressed genes and pathways for intramuscular fat deposition in pectoralis major tissues of fast-and slow-growing chickens. *BMC Genomics*. 2012;13(1):213. 22646994 10.1186/1471-2164-13-213 PMC3420248

[2] Liu X, Wang C, Wang Y, et al. Age-associated changes in the growth development of abdominal fat and their correlations with cecal gut microbiota in broiler chickens. *Poult Sci*. 2023;102(9):102900. 37406441 10.1016/j.psj.2023.102900 PMC10466292

[3] Nematbakhsh S, Pei PC, Selamat J, et al. Molecular regulation of lipogenesis, adipogenesis and fat deposition in chicken. *Genes (Basel)*. 2021;12(3):414. 33805667 10.3390/genes12030414 PMC8002044

[4] Han Q, Huang X, He J, et al. Intramuscular fat deposition in pig: a key target for improving pork quality. *J. Integr. Agr*. 2025;24(12):4461–4483.

[5] Fu R, Liu R, Zhao G, et al. Expression profiles of key transcription factors involved in lipid metabolism in Beijing-You chickens. *Gene*. 2014;537(1):120–125. 24100085 10.1016/j.gene.2013.07.109

[6] Na W, Wu Y, Gong P, et al. Embryonic transcriptome and proteome analyses on hepatic lipid metabolism in chickens divergently selected for abdominal fat content. *BMC Genomics*. 2018;19(1):384. 29792171 10.1186/s12864-018-4776-9 PMC5966864

[7] Mancinelli A, Veroli A, Mattioli S, et al. Lipid metabolism analysis in liver of different chicken genotypes and impact on nutritionally relevant polyunsaturated fatty acids of meat. *Sci Rep-Uk.* 2022;12:1888. 10.1038/s41598-022-05986-2 PMC8814176 35115659

[8] Gibbons GF, Islam K, Pease RJ. Mobilisation of triacylglycerol stores. *Biochim Biophys Acta*. 2000;1483(1):37–57. 10601694 10.1016/s1388-1981(99)00182-1

[9] Xu W, Iwasawa A, Yayota M. Effects of early experience with low-quality roughage on liver metabolome in lambs. *Metabolomics.* 2017;13(8):90.

[10] Cortes M, Pareja E, García-Cañaveras JC, Donato, et al. Metabolomics discloses donor liver biomarkers associated with early allograft dysfunction. *J Hepatol*. 2014;61(3):564–574. 24798621 10.1016/j.jhep.2014.04.023

[11] Hirai MY, Yano M, Goodenowe DB, et al. Integration of transcriptomics and metabolomics for understanding of global responses to nutritional stresses in Arabidopsis thaliana. *Proc Natl Acad Sci U S A*. 2004;101(27):10205–10210. 15199185 10.1073/pnas.0403218101 PMC454188

[12] Tang Y, Yin L, Liu L, et al. Comparative analysis of different proteins and metabolites in the liver and ovary of local breeds of chicken and commercial chickens in the later laying period. *Int J Mol Sci*. 2023;24(18):14394. 37762699 10.3390/ijms241814394 PMC10531955

[13] Xu L, Liu Z, Gong M, et al. Transcriptome and metabolite profiling reveals the mechanism of hepatic lipid metabolism during fasting in chicken. *Anim Biosci*. 2026;39(1):250014. 40808561 10.5713/ab.25.0014 PMC12754495

[14] Li H, Li Y, Yang L, et al. Identification of a novel lipid metabolism-associated hepatic gene family induced by estrogen via ERα in chicken (Gallus gallus). *Front Genet*. 2020;11:271. 32296460 10.3389/fgene.2020.00271 PMC7136477

[15] Gao L, Yuan H, Xu E, et al. Toxicology of paraquat and pharmacology of the protective effect of 5-hydroxy-1-methylhydantoin on lung injury caused by paraquat based on metabolomics. *Sci Rep*. 2020;10(1):1790. 32019966 10.1038/s41598-020-58599-y PMC7000692

[16] Rui L. Energy metabolism in the liver. *Compr Physiol*. 2014;4(1):177–197. 24692138 10.1002/cphy.c130024 PMC4050641

[17] Li H, Wang T, Xu C, et al. Transcriptome profile of liver at different physiological stages reveals potential mode for lipid metabolism in laying hens. *BMC Genomics*. 2015;16(1):763. 26452545 10.1186/s12864-015-1943-0 PMC4600267

[18] Cochrane AW, Deeley RG. Estrogen-dependent activation of the avian very low density apolipoprotein II and vitellogenin genes. Transient alterations in mRNA polyadenylation and stability early during induction. *J Mol Biol*. 1988;203(3):555–567. 3210227 10.1016/0022-2836(88)90192-1

[19] Cui Z, Amevor FK, Feng Q, et al. Sexual maturity promotes yolk precursor synthesis and follicle development in hens via liver-blood-ovary signal axis. *Animals (Basel)*. 2020;10(12):2348. 33317071 10.3390/ani10122348 PMC7763865

[20] He Q, Ren P, Kong X, et al. Comparison of serum metabolite compositions between obese and lean growing pigs using an NMR-based metabonomic approach. *J Nutr Biochem*. 2012;23(2):133–139. 21429726 10.1016/j.jnutbio.2010.11.007

[21] Ren P, Chen M, Liu Q, et al. Gga-let-7a-3p inhibits the proliferation and differentiation of chicken intramuscular preadipocytes. *Br Poult Sci*. 2024;65(1):34–43. 37807894 10.1080/00071668.2023.2264807

[22] Li J, Huang Q, Yang C, et al. Molecular regulation of differential lipid molecule accumulation in the intramuscular fat and abdominal fat of chickens. *Genes (Basel)*. 2023;14(7):1457. 37510361 10.3390/genes14071457 PMC10379444

[23] Longo N, Frigeni M, Pasquali M. Carnitine transport and fatty acid oxidation. *Biochim Biophys Acta*. 2016;1863(10):2422–2435. 26828774 10.1016/j.bbamcr.2016.01.023 PMC4967041

[24] Carlson DB, McFadden JW, D’Angelo A, et al. Dietary L-carnitine affects periparturient nutrient metabolism and lactation in multiparous cows. *J Dairy Sci*. 2007;90(7):3422–3441. 17582127 10.3168/jds.2006-811

[25] Kathirvel E, Morgan K, French SW, et al. Acetyl-L-carnitine and lipoic acid improve mitochondrial abnormalities and serum levels of liver enzymes in a mouse model of nonalcoholic fatty liver disease. *Nutr Res*. 2013;33(11):932–941. 24176233 10.1016/j.nutres.2013.08.001

[26] Wang X, Han W, Yang J, et al. Development of chemical isotope labeling LC-MS for tissue metabolomics and its application for brain and liver metabolome profiling in Alzheimer’s disease mouse model. *Anal Chim Acta*. 2019;1050:95–104. 30661596 10.1016/j.aca.2018.10.060

[27] Holeček M. Histidine in health and disease: metabolism, physiological importance, and use as a supplement. *Nutrients*. 2020;12(3):848. 32235743 10.3390/nu12030848 PMC7146355

[28] Novak C, Yakout HM, Scheideler SE. The effect of dietary protein level and total sulfur amino acid: lysine ratio on egg pro-duction parameters and egg yield in Hy-Line W-98 hens. *Poult Sci*. 2006;85(12):2195–2206. 17135677 10.1093/ps/85.12.2195

[29] Nie C, Zhang W, Wang Y, et al. Tissue lipid metabolism and hepatic metabolomic profiling in response to supplementation of fermented cottonseed meal in the diets of broiler chickens. *J Zhejiang Univ Sci B*. 2015;16(6):447–455. 26055906 10.1631/jzus.B1400255 PMC4471596

[30] Xian L, Wang Y, Peng D, et al. Dietary oregano oil supplementation improved egg quality by altering cecal microbiota function in laying hens. *Animals (Basel)*. 2024;14(22):3235. 39595288 10.3390/ani14223235 PMC11591137

[31] Massudi H, Grant R, Guillemin GJ, et al. NAD+ metabolism and oxidative stress: the golden nucleotide on a crown of thorns. *Redox Rep*. 2012;17(1):28–46. 22340513 10.1179/1351000212Y.0000000001 PMC6837626

[32] Yin F, Boveris A, Cadenas E. Mitochondrial energy metabolism and redox signaling in brain aging and neurodegeneration. *Antioxid Redox Signal*. 2014;20(2):353–371. 22793257 10.1089/ars.2012.4774 PMC3887431

[33] Clement J, Wong M, Poljak A, et al. The plasma NAD(+) metabolome is dysregulated in “Normal” aging. *Rejuvenation Res*. 2019;22(2):121–130. 30124109 10.1089/rej.2018.2077 PMC6482912

[34] Kim SE, Jin DK, Cho SS, et al. Regional cerebral glucose metabolic abnormality in Prader-Willi syndrome: a 18F-FDG PET study under sedation. *J Nucl Med*. 2006;47(7):1088–1092. 16818941

[35] Rengaraj D, Lee SI, Yoo M, et al. Expression and knockdown analysis of glucose phosphate isomerase in chicken primordial germ cells. *Biol Reprod*. 2012;87(3):57. 22699485 10.1095/biolreprod.112.101345

[36] Rajas F, Gautier-Stein A, Mithieux G. Glucose-6 phosphate, a central hub for liver carbohydrate metabolism. *Metabolites*. 2019;9(12):282. 31756997 10.3390/metabo9120282 PMC6950410

[37] Cai Y, Song Z, Zhang X, et al. Increased de novo lipogenesis in liver contributes to the augmented fat deposition in dexamethasone exposed broiler chickens (Gallus gallus domesticus). *Comp Biochem Physiol C Toxicol Pharmacol*. 2009;150(2):164–169. 19393339 10.1016/j.cbpc.2009.04.005

[38] Stachowiak M, Nowacka-Woszuk J, Szydlowski M, et al. The ACACA and SREBF1 genes are promising markers for pig carcass and performance traits, but not for fatty acid content in the longissimus dorsi muscle and adipose tissue. *Meat Sci*. 2013;95(1):64–71. 23657179 10.1016/j.meatsci.2013.04.021

[39] Matsumoto H, Sasaki K, Bessho T, et al. The SNPs in the ACACA gene are effective on fatty acid composition in Holstein milk. *Mol Biol Rep*. 2012;39(9):8637–8644. 22718502 10.1007/s11033-012-1718-5

[40] Du X, Lai S, Zhao W, et al. Single-cell RNA sequencing revealed the liver heterogeneity between egg-laying duck and ceased-laying duck. *BMC Genomics*. 2022;23(1):857. 36577943 10.1186/s12864-022-09089-0 PMC9798604

[41] Richards MP, Poch SM, Coon CN, et al. Feed restriction significantly alters lipogenic gene expression in broiler breeder chickens. *J Nutr*. 2003;133(3):707–715. 12612141 10.1093/jn/133.3.707

[42] Sánchez-Gurmaches J, Cruz-Garcia L, Gutiérrez J, et al. mRNA expression of fatty acid transporters in rainbow trout: in vivo and in vitro regulation by insulin, fasting and inflammation and infection mediators. *Comp Biochem Physiol A Mol Integr Physiol*. 2012;163(2):177–188. 22771331 10.1016/j.cbpa.2012.06.010

[43] Wang Z, Yue Y, Liu Z, et al. Genome-wide analysis of the FABP gene family in liver of chicken (gallus gallus): identification, dynamic expression profile, and regulatory mechanism. *Int J Mol Sci*. 2019;20(23):5948. 31779219 10.3390/ijms20235948 PMC6928644

[44] Li Q, Wang C, Li A, et al. Genetic variants affecting FADS2 enzyme dynamics and gene expression in cogenetic oysters with different PUFA levels provide new tools to improve unsaturated fatty acids. *Int J Mol Sci*. 2024;25(24):13551. 39769316 10.3390/ijms252413551 PMC11677070

[45] Witt A, Mateska I, Palladini A, et al. Fatty acid desaturase 2 determines the lipidomic landscape and steroidogenic function of the adrenal gland. *Sci Adv*. 2023;9(29):eadf6710. 37478183 10.1126/sciadv.adf6710 PMC10361602

[46] Zhang Y, Luo PY, Tang YN, et al. Association between the non-high-density lipoprotein cholesterol to high-density lipoprotein cholesterol ratio (NHHR) and mortality in patients with metabolic dysfunction-associated steatotic liver disease (MASLD): data from the NHANES III (1988–1994). *Nutr Metab (Lond)*. 2025;22(1):46. 40399925 10.1186/s12986-025-00942-z PMC12093885

[47] Yang K, Shi R, Yang S, et al. Effects of fetuin and glycolipid metabolism on metabolic associated fatty liver disease. *Advances in Clinical Medicine.* 2022;12:7360–7366.

[48] Sassa T, Tadaki M, Kiyonari H, et al. Very long-chain tear film lipids produced by fatty acid elongase ELOVL1 prevent dry eye disease in mice. *Faseb J*. 2018;32(6):2966–2978. 29401594 10.1096/fj.201700947R

[49] Cardoso L, Cecatto C, Ozola M, et al. Fatty acid β-oxidation in brain mitochondria: Insights from high-resolution respirometry in mouse, rat and Drosophila brain, ischemia and aging models. *Biochim Biophys Acta Mol Basis Dis*. 2025;1871(1):167544. 39424161 10.1016/j.bbadis.2024.167544

[50] Li Z, Zhang B, Liu Q, et al. Genetic association of lipids and lipid-lowering drug target genes with non-alcoholic fatty liver disease. *EBioMedicine*. 2023;90:104543. 37002989 10.1016/j.ebiom.2023.104543 PMC10070091

[51] Sun S, Liu Y, Limbu S, et al. Vitellogenin 1 is essential for fish reproduction by transporting DHA-containing phosphatidylcholine from liver to ovary. *Biochim Biophys Acta Mol Cell Biol Lipids*. 2023;1868(4):159289. 36708962 10.1016/j.bbalip.2023.159289

