# Supplementary figures and images for: Integrative metabolomic and transcriptomic analysis reveals stage-specific shifts in hepatic lipid metabolism of broiler chickens
Source: Anim Biotechnol. 2026 Feb 3;37(1):2622124. doi: 10.1080/10495398.2026.2622124 (PMC12875106; doi:10.1080/10495398.2026.2622124)

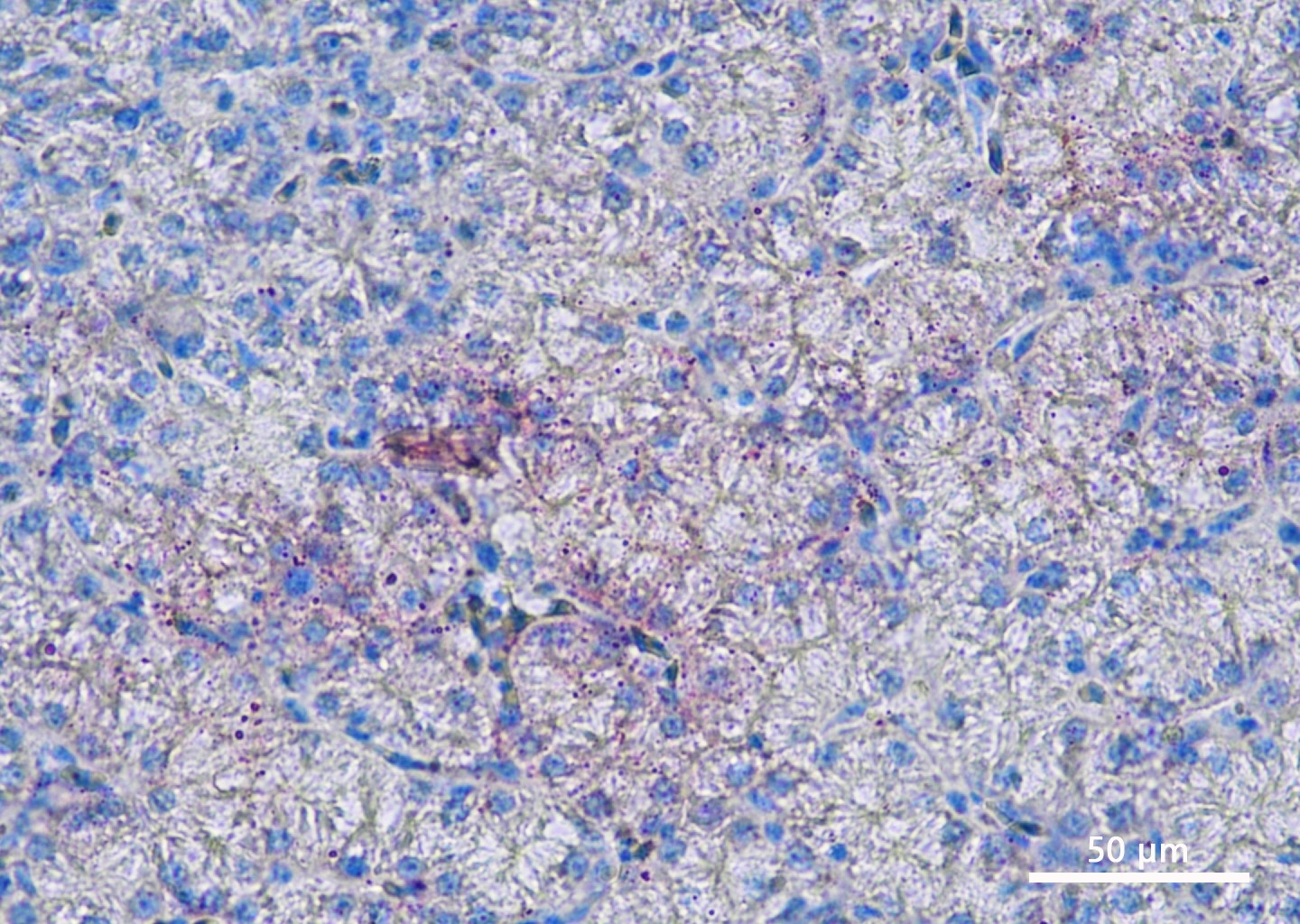

Supplement: Original Image for Fig 1G.jpeg [file LABT_A_2622124_SM2142.jpeg]

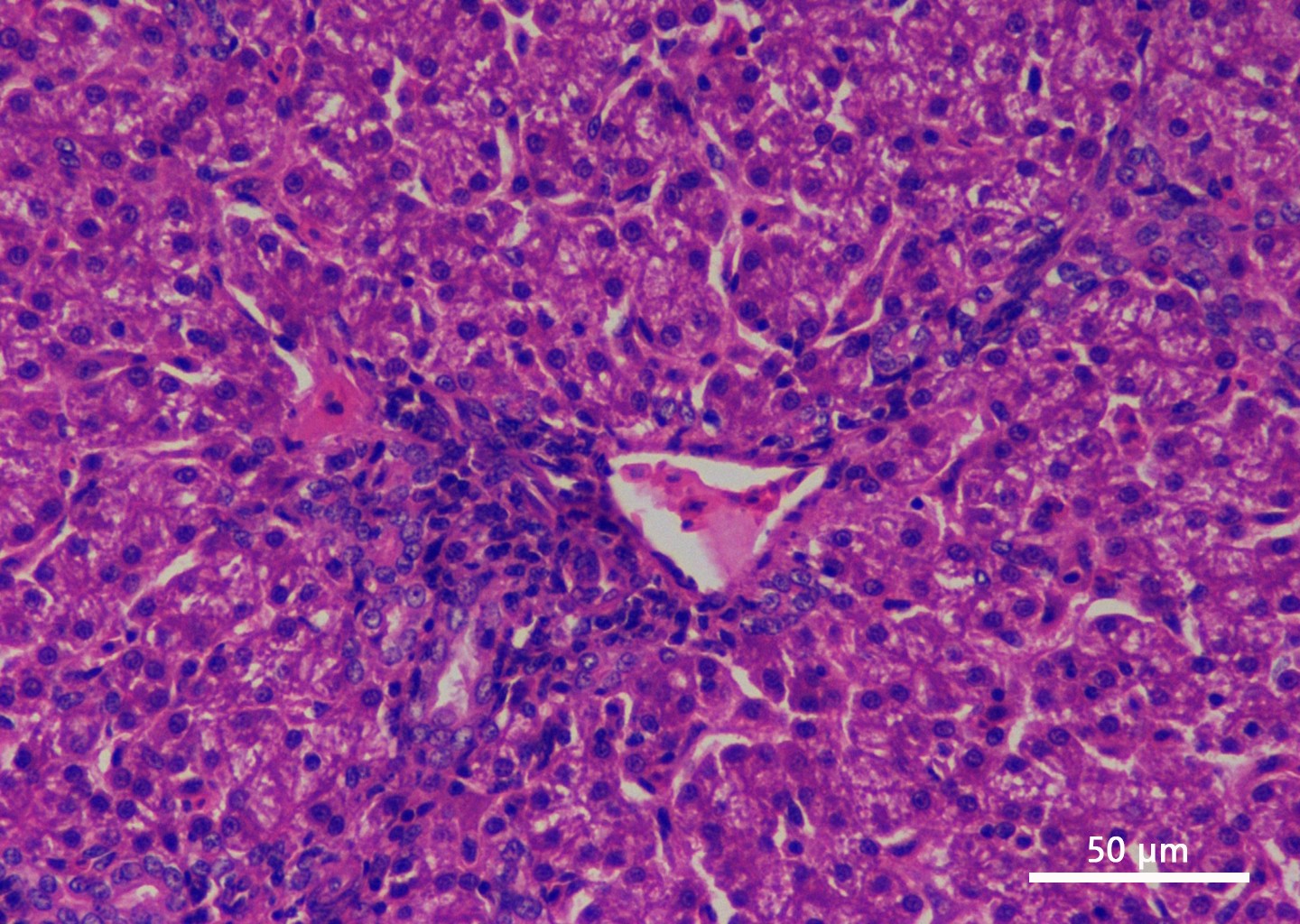

Supplement: Original Image for Fig 1C.jpeg [file LABT_A_2622124_SM2141.jpeg]

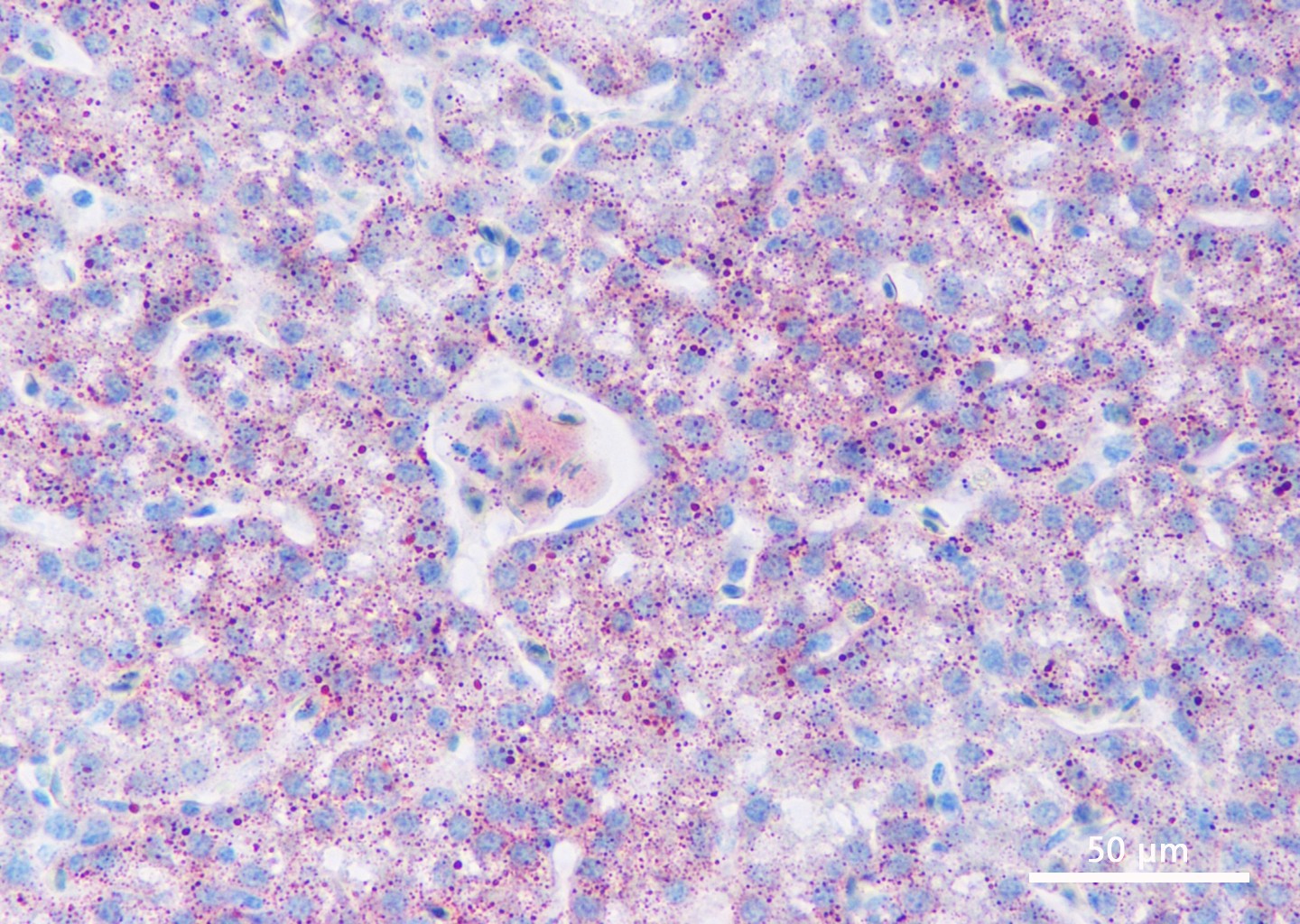

Supplement: Original Image for Fig 1I.jpeg [file LABT_A_2622124_SM2140.jpeg]

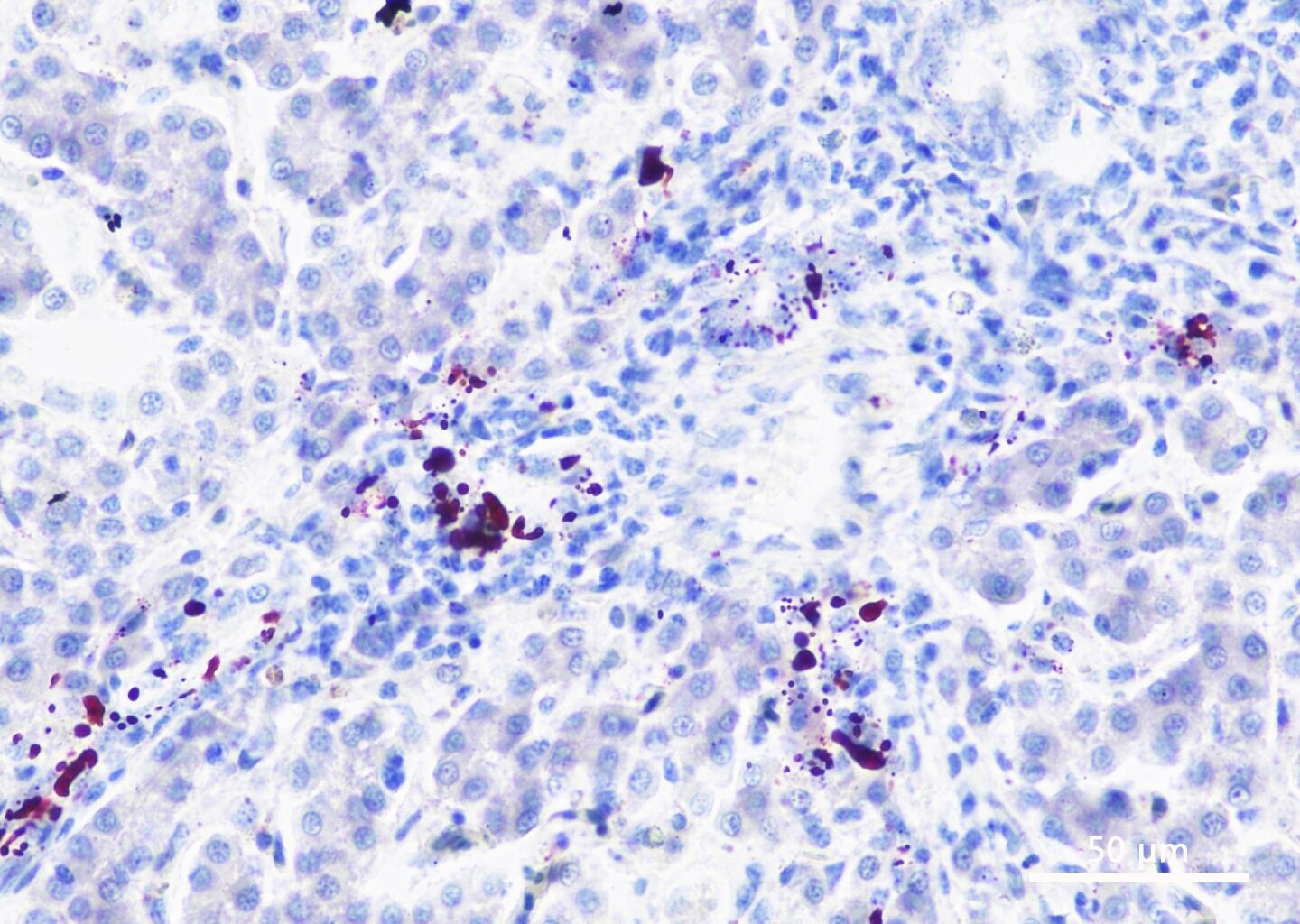

Supplement: Original Image for Fig 1H.jpeg [file LABT_A_2622124_SM2139.jpeg]

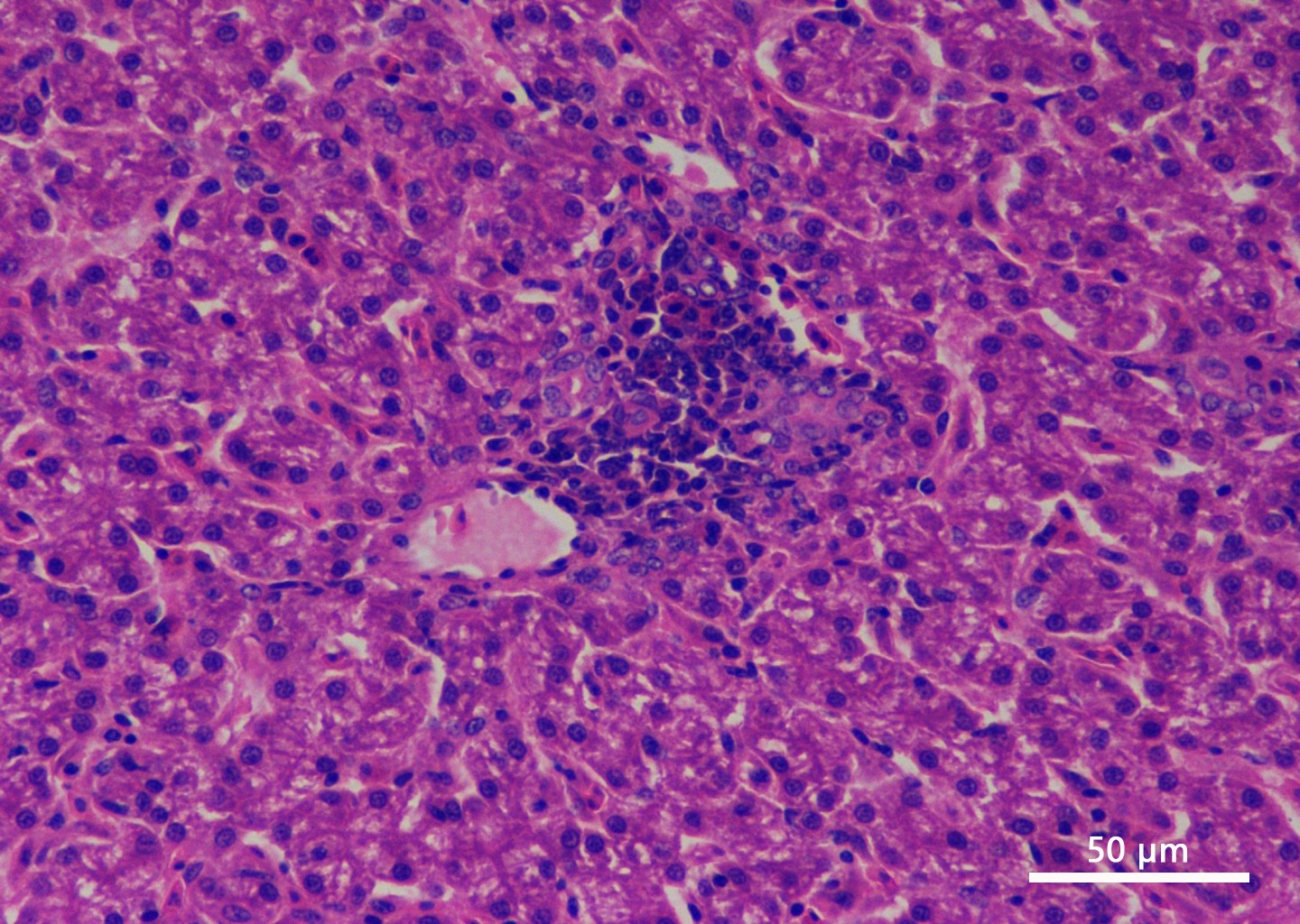

Supplement: Original Image for Fig 1B.jpeg [file LABT_A_2622124_SM2138.jpeg]

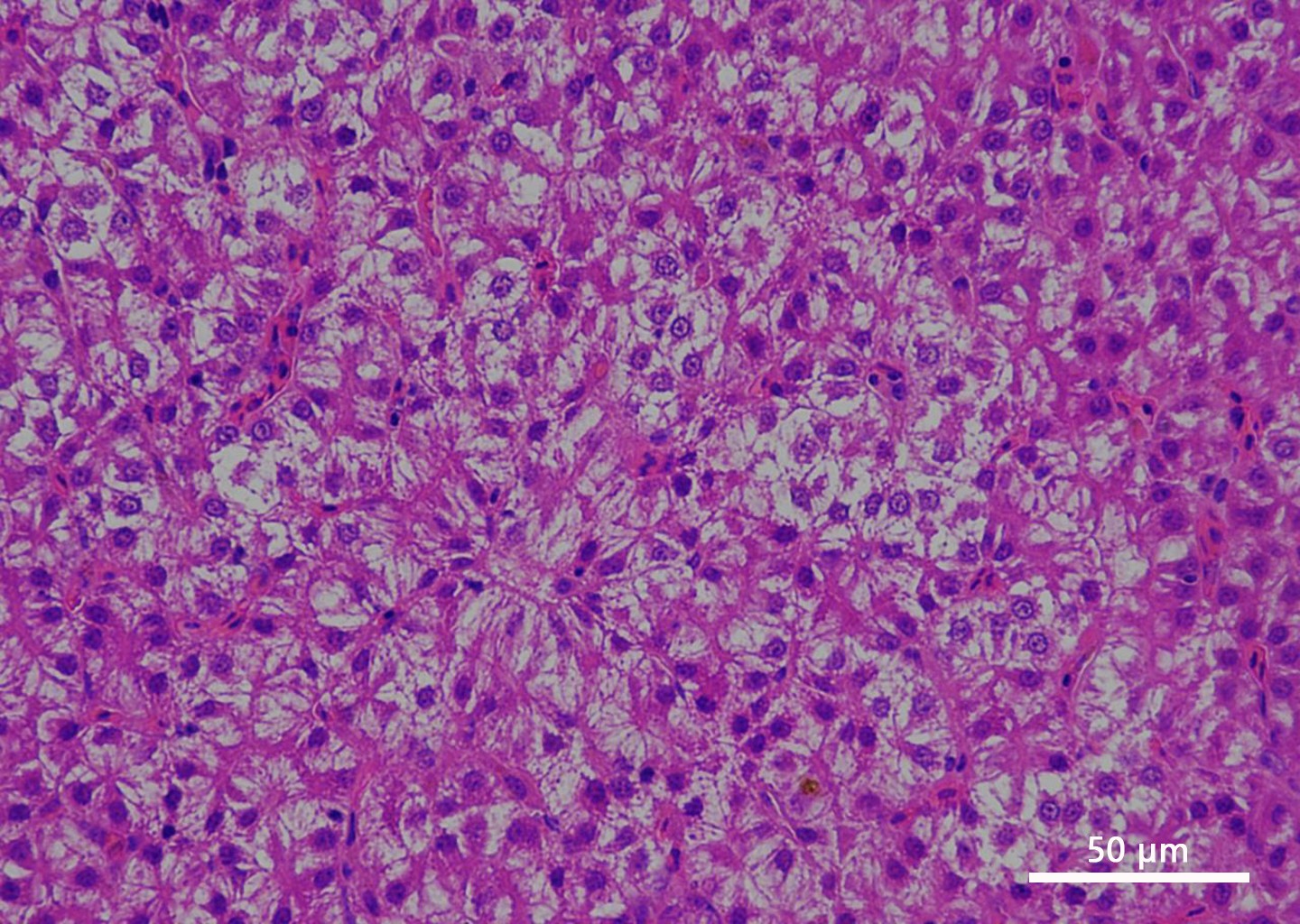

Supplement: Original Image for Fig 1E.jpeg [file LABT_A_2622124_SM2137.jpeg]

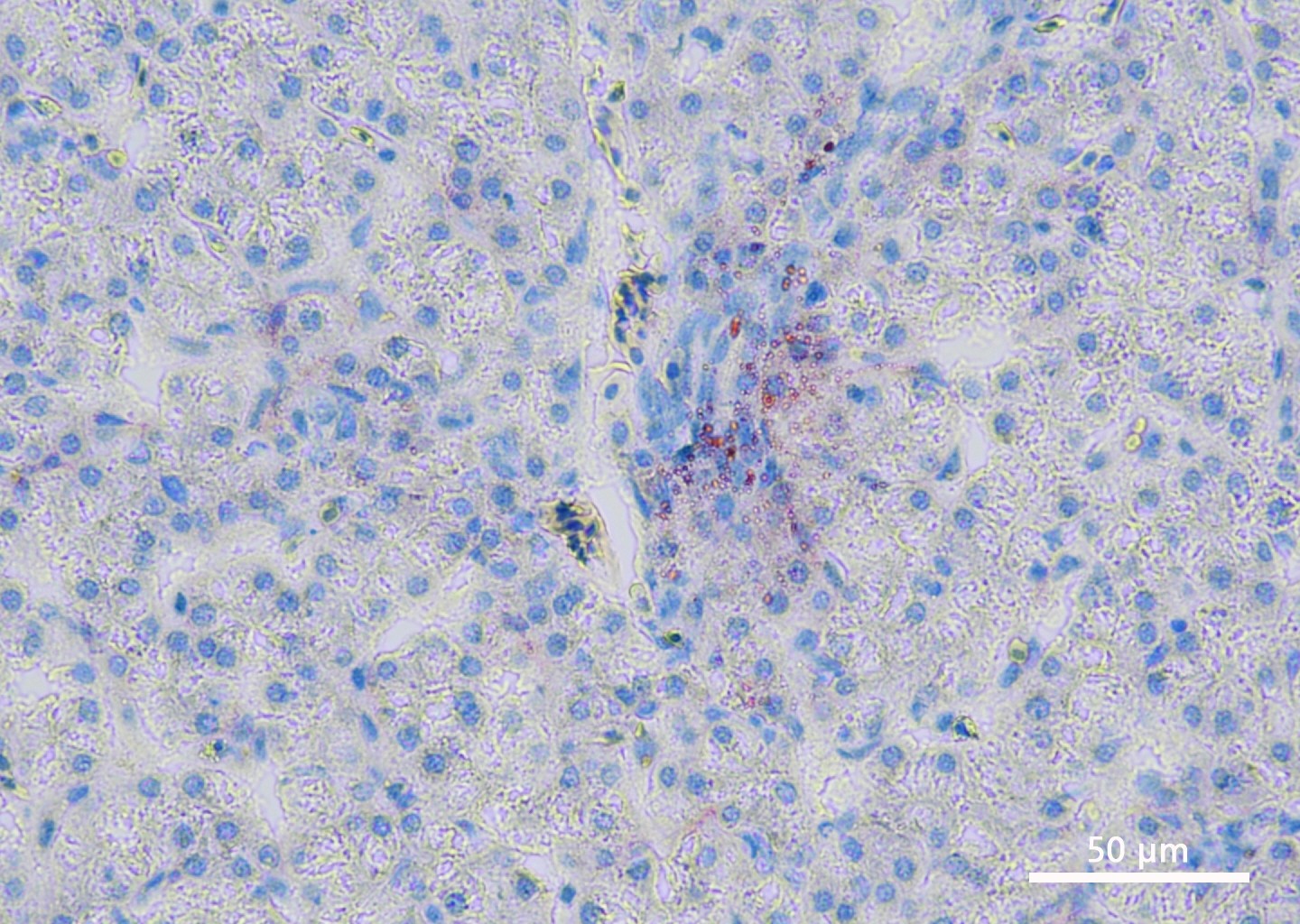

Supplement: Original Image for Fig 1F.jpeg [file LABT_A_2622124_SM2136.jpeg]

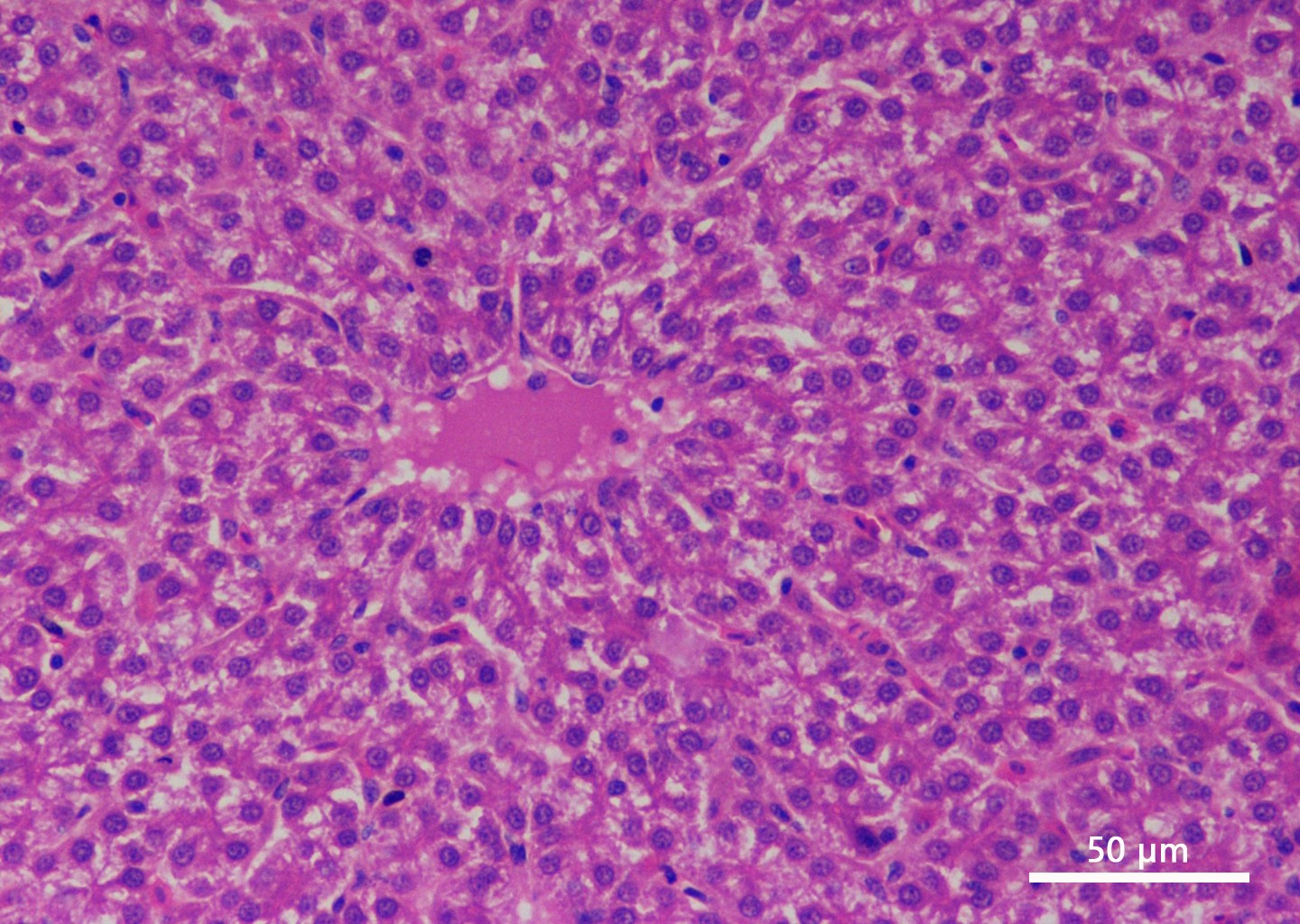

Supplement: Original Image for Fig 1D.jpeg [file LABT_A_2622124_SM2134.jpeg]

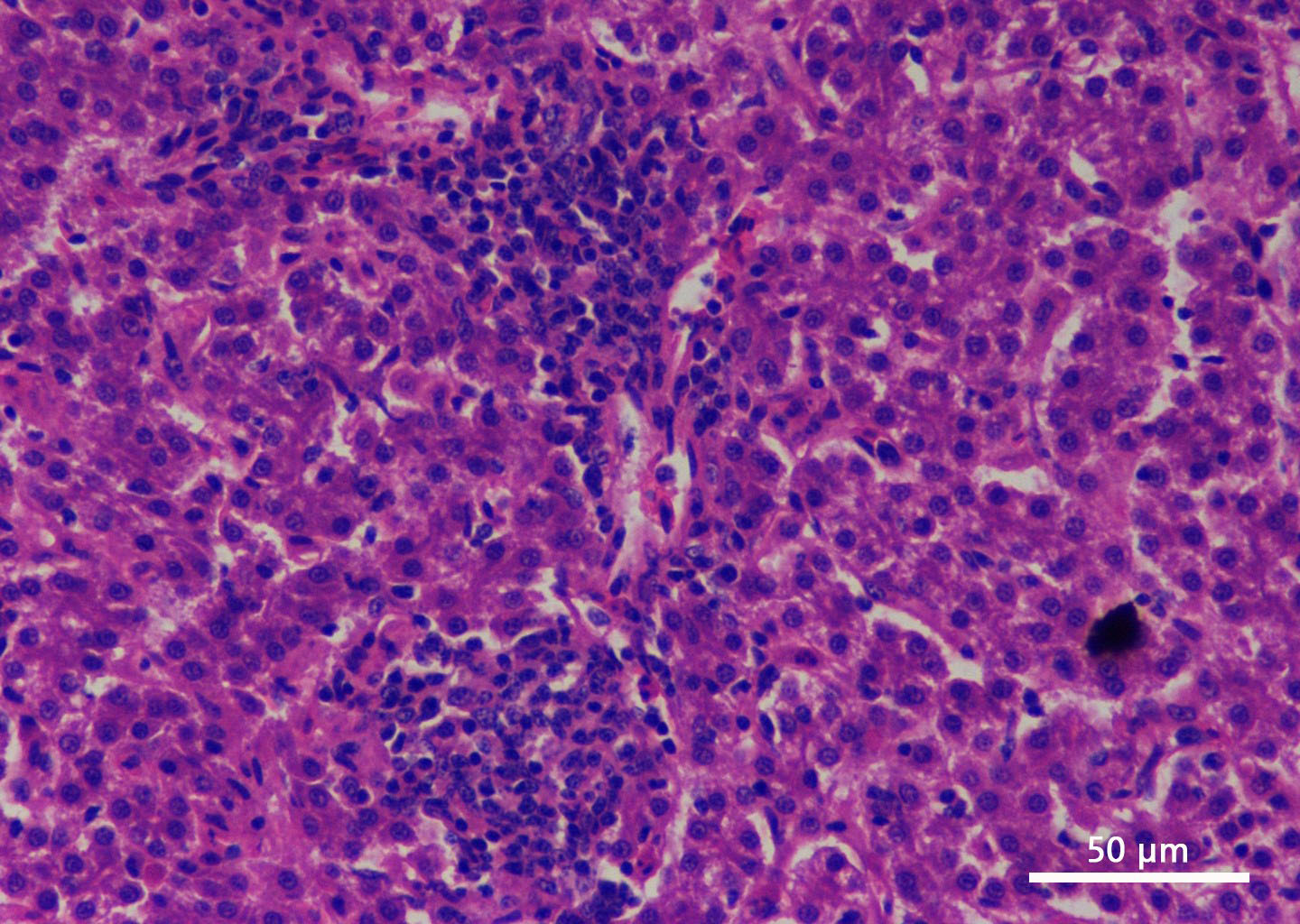

Supplement: Original Image for Fig1A.tif [file LABT_A_2622124_SM2133.tif]

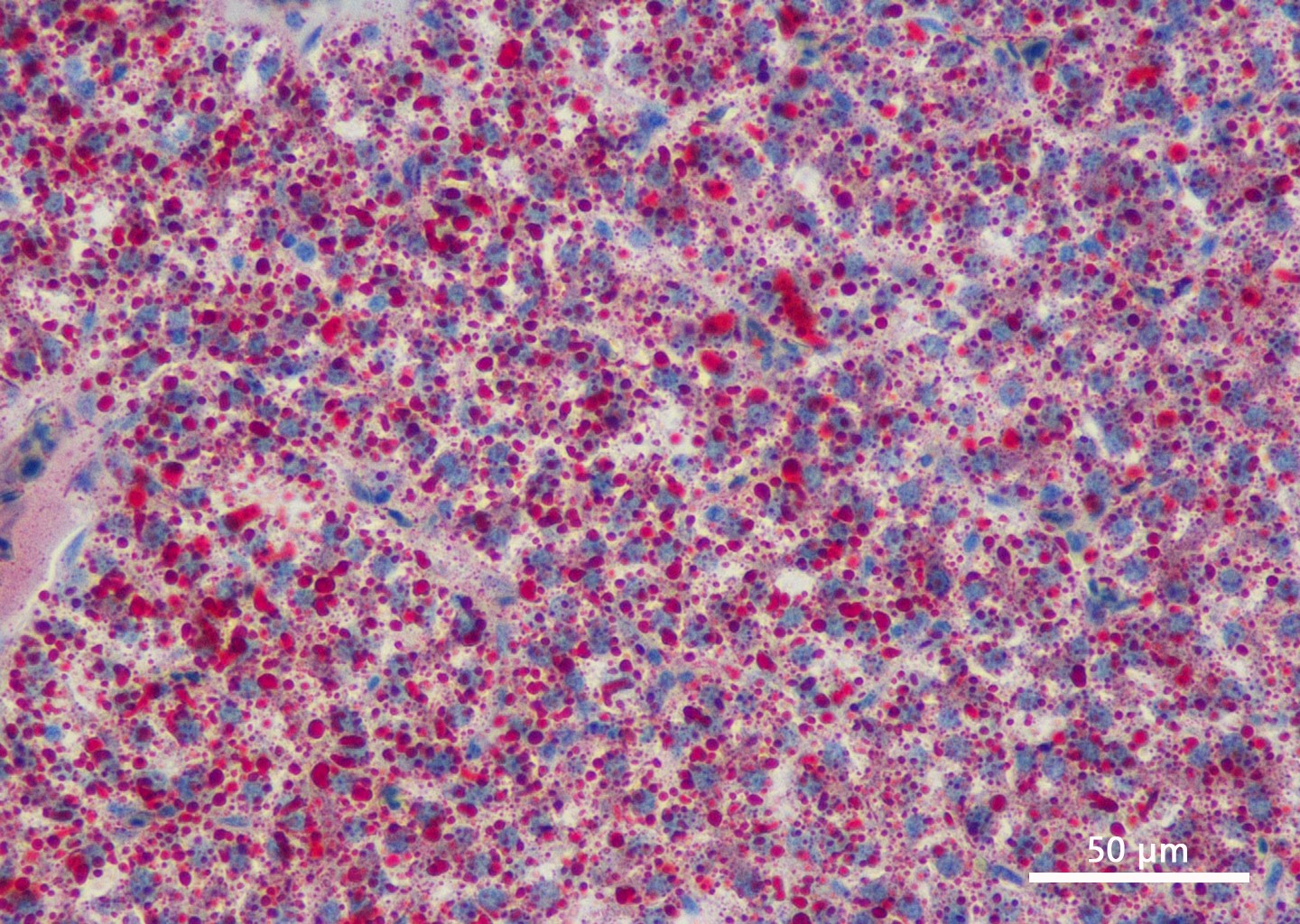

Supplement: Original Image for Fig 1J.jpeg [file LABT_A_2622124_SM2132.jpeg]
