# Supplementary material for: Integrative metabolomic and transcriptomic analysis reveals stage-specific shifts in hepatic lipid metabolism of broiler chickens
Source: Anim Biotechnol. 2026 Feb 3;37(1):2622124. doi: 10.1080/10495398.2026.2622124 (PMC12875106; doi:10.1080/10495398.2026.2622124)
Supplement: suplementary materials.docx [file LABT_A_2622124_SM2135.docx]

**Supplementary Figures**

**Supplementary Fig. S1.** (A-D) The permutation testing of d 60 vs. d 90, d 90 vs. d 120, d 120 vs. d 150, and d 150 vs. d 180, respectively. (E) The permutation among the 5 age groups.

**Supplementary Fig. S2.** Trend analysis of identified metabolites

**Supplementary Fig. S1:**





**Supplementary Fig. S2:**


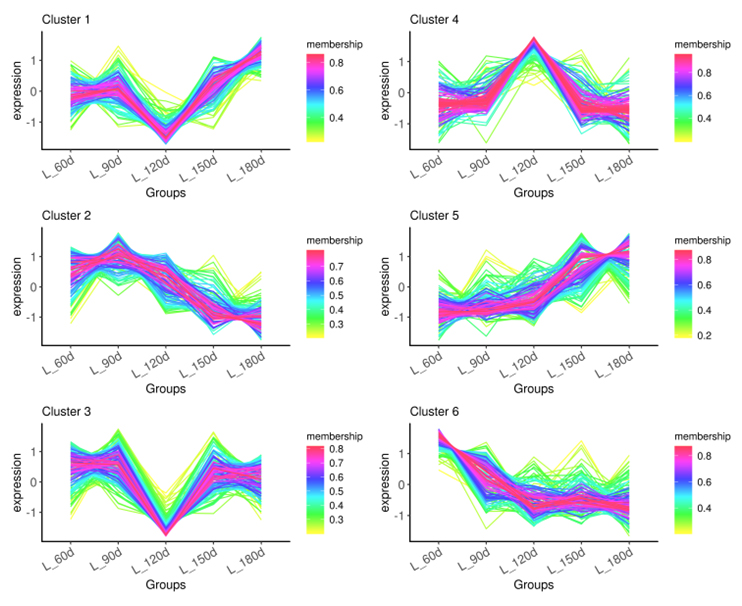


**Supplementary Tables**

Supplementary Table S1. The specific primers for qPCR in this study

| Accession NO. | Gene  symbol | | Primer sequence | | Product size | |
| --- | --- | --- | --- | --- | --- | --- |
| NM_001305183.2 | *ADH1C* | | F: AGCTTGACTCGGCACAAGAA  R: CATCAGAGCGACAGATCCCC | | 211 | |
| XM_015286122.4 | *TKFC* | | F: CGTGGTGACAGTGGACAGAA  R: ATGCCCTTCCCAATGTAGCC | | 270 | |
| NM_001044633.2 | *APOB* | | F: GGTTACTCCCACGATGGCAA | | 120 | |
|  |  |  | R: TCGCAGAAATGCCCTTCCTT | |  |  |
| XM_046904333.1 | *PHOSPHO1* | | F: TAGCTCCCATTGCCCTCACC  R: TGGGGGAGGAAGAAGAAGGT | | 119 | |
| NM_001031420.2 | | | *PLIN2* | F: TCTTTGCGGGCTCTGTAAGAT  R: ACCCTCGACACGATGTTCTG | 83 | |
| XM_046942379.1 | | | *FADS1* | F: GACTCCCTGGCTCTGTTCTC  R: CAGGGAAGTGCCACTGATAGAG | 123 | |
| NM_001160428.3 | *FADS2* | | F: CAACAATGCCACGGCACAA  R: TGACCTGTTCCCTCCCCATT | | 201 | |
| XM_015294080.4 | *ACSL6* | | F: TCAGGGAGAGATGGAATCGC  R: GCCTTCGGTCCGTGAAATTG | | 258 | |
| XM_046943363.1 | *GPAM* | | F: CATGCTCACTGGGGTCTTTTG  R: GTTTGGTCATAGACAGGGGCA | | 151 | |
| XM_040702445.2 | *ACSL5* | | F: AGAGTTCCAGAAAATGAGCCATCA  R: ATTGCTCTCCTATCCCCTCCT | | 295 | |
| NM_204192.4 | *FABP1* | | F: CAGGAGAGAAGGCCAAGTGT  R: CACGGATTTCAGCCCCTTCA | | 80 | |
| NM_001197308.2 | *ELOVL2* | | F: GTGCCGAGGAGACCTTACTG  R: GTGCCAGTGCTTCACCTACT | | 187 | |
| NM_204420.3 | *MMP2* | | F: CGATGCTGTCTACGAGTCCC  R: TAGCCCCTATCCAGGTTGCT | | 96 | |
| NM_001030889.2 | *FABP3* | | F: CCGCACCTATGAGAAGGCAT  R: CCCCATGAGACCACAGCATC | | 165 | |
| XM_015276089.4 | *LPIN1* | | F: TCCAGCAGATGGAGGCCTAT  R: GCACTCTGGATGACCCGAAA | | 279 | |
| XM_015277252.4 | *HMGCS1* | | F: GTGAGGGAGGAGAGGAGGG  R: GTTGGGGTTTGTTCGCATCC | | 279 | |
| XM_015276582.4 | *ETNPPL* | | F: GAACTTCTTAAGTCCGTGCCG  R: CTCCTCCGCATCCCCAAAAG | | 82 | |
| XM_046905271.1 | *LPL* | | F: AGCAAAAGGTAACAGCTGACAA  R: GCGTCCTCGCAAGAAGCAA | | 236 | |
| NM_205525.5 | *APOA1* | | F: CTACAAGGAGGTGCGCGAGA  R: CCGTCCACTTGGCAGAGAACT | | 128 | |
| NM_001398307.1 | *VTG3* | | F: TTGCTCAGTCGTGGGTCATC  R: GGAACAGTCCTGGTTGCTGA | | 172 | |
| NM_001004408.3 | *VTG1* | | F: AGCTCTGGTGTTTCCCATCG  R: GTCCTGTGTCCTGAATGCCA | | 182 | |
| NM_204290.2 | *FABP4* | | F: CTGGCCTGACAAAATGTGCG  R: CCACACCAGCCATCTTCCTG | | 122 | |
| XM_046899058.1 | *PLIN1* | | F: CAATGCCACAATCACAGCACCC  R: TTGAGACTGCCTTGGCCCTT | | 195 | |
| NM_205155.4 | *FASN* | | F: GCGTGAACTGTCCTCCAAGA  R: TCGTTGGCCCTTCTGGATTC | | 128 | |
| XM_046942569.1 | *PPARA* | | F: AACCGTCCTTCAAAATGCGG  R: ACAACATCCTTTTCCGGGCA | | 103 | |
| NM_001031145.2 | *GPAT3* | | F: TCTCGTTGGTATTCGCCCTC  R: CATGTAGGCCTCGGAGATGC | | 74 | |
| XM_040650094.2 | *ACACA* | | F: GGAAGTCGGGAGCGTACCA  R: GCAGTCGTGGCTGAAATCCT | | 70 | |
| XM_015287125.4 | *ACOT1L* | | F: TTGGGAACTGTCTTGCCCC  R: TGTCACCATGCTGGGTCTTC | | 94 | |
| XM_046905334.1 | *APOV1* | | F: CAGTGCAGGATACTGAGCGTTA  R: AAGATGGGGTCCTTGCAGAAG | | 133 | |
| XM_046939500.1 | *MBOAT2* | | F: ACTGCCTCCAACTTCACCAC  R: AATTCGGCAACATCAGCGTT | | 104 | |
| XM_046922758.1 | *ELOVL1* | | F: TGGAGGATGGATCAGGTCGT  R: GTGTACCCAGTAAGCCACCC | | 156 | |
| NM_001006164.2 | *PEMT* | | F: CCAGCAACCCCTAATGAGCC  R: TGGAGCTGGGTTGCTTCATC | | 161 | |
| NM_205505.2 | *ACACA* | | F: AATGGCAGCTTTGGAGGTGT  R: TTCTGTTTGGGTGGGAGGTG | | 137 | |
| NM_205294.2 | *RXRG* | | F: CTCTTCAACCCAGACGCCAA  R: TGCTTCGTGTAGGCTTCCAG | | 95 | |
| XM_046936177.1 | *ALDH7A1* | | F: TCGTGGTATCCCTGCACCTA  R: TTTCTAGGGTGCGTGTGGTC | | 169 | |

Supplementary Table S2. Significantly differential metabolites between age groups annotated to “lipids and lipid-like molecules” in HMDB or Lipid Maps database

| Significant unique  metabolites | Groups | FC | VIP | P  value | Trend | Identifier from relevant database |
| --- | --- | --- | --- | --- | --- | --- |

| Myricetin | 120vs90 | 2.244 | 1.910 | 0.001 | up | LMPK12110001 |
| --- | --- | --- | --- | --- | --- | --- |
|  | 150vs120 | 0.529 | 1.433 | 0.004 | down | HMDB0002755；LMPK12110001 |
| LPI 22:6 | 90vs60 | 0.441 | 1.549 | 0.000 | down | LMGP06050012 |
|  | 150vs120 | 4.499 | 1.886 | 0.000 | up | LMGP06050012 |
| 2-Isopropylmalate | 120vs90 | 1.663 | 1.426 | 0.004 | up | HMDB0000402 |
| LPE 18:3 | 90vs60 | 0.613 | 1.300 | 0.029 | down | LMGP02050043 |
|  | 120vs90 | 0.505 | 1.239 | 0.006 | down | LMGP02050043 |
| PC (20:1/20:1) | 180vs150 | 1.550 | 1.418 | 0.011 | up | LMGP01011043 |
| LPI 20:4 | 150vs120 | 2.565 | 1.747 | 0.000 | up | LMGP06050006 |
| Nonadecanoic acid | 90vs60 | 1.916 | 1.856 | 0.016 | up | HMDB0000772；LMFA01010019 |
| Eicosapentaenoic acid | 120vs90 | 0.352 | 1.826 | 0.000 | down | HMDB0001999 |
|  | 180vs150 | 1.767 | 1.307 | 0.048 | up | HMDB0001999 |
| 13-OxoODE | 120vs90 | 0.398 | 1.240 | 0.020 | down | HMDB0004668；LMFA02000252 |
|  | 150vs120 | 1.993 | 1.197 | 0.001 | up | HMDB0004668；LMFA02000252 |
| LPG 16:0 | 180vs150 | 2.093 | 2.354 | 0.010 | up | LMGP04050008 |
| Docosatrienoic acid | 150vs120 | 0.274 | 1.580 | 0.000 | down | HMDB0002823 |
|  | 90vs60 | 1.742 | 1.560 | 0.034 | up | HMDB0014368 |
|  | 120vs90 | 0.599 | 1.046 | 0.048 | down | HMDB0014368 |
| alpha-Farnesene | 90vs60 | 0.655 | 1.377 | 0.008 | down | HMDB0036065 |
|  | 120vs90 | 0.521 | 1.336 | 0.002 | down | HMDB0036065 |
|  | 150vs120 | 2.492 | 1.708 | 0.000 | up | HMDB0036065 |
| Pregnenolone | 120vs90 | 0.291 | 1.356 | 0.004 | down | HMDB0000253；LMST02030088 |
|  | 150vs120 | 3.576 | 1.530 | 0.000 | up | HMDB0000253；LMST02030088 |
| Pentadecanoic acid | 150vs120 | 3.569 | 1.015 | 0.015 | up | HMDB0000826；LMFA01010015 |
| L-Palmitoylcarnitine | 90vs60 | 4.256 | 2.089 | 0.006 | up | HMDB0000222；LMFA07070079 |
| Estrone | 150vs120 | 1.800 | 1.220 | 0.034 | up | HMDB0000145；LMST02010004 |
| 8(S)-Hydroxy-(5Z,9E,11Z,14Z)-eicosatetraenoic acid | 90vs60 | 0.480 | 1.679 | 0.037 | down | HMDB0004679 |
|  | 150vs120 | 2.342 | 1.492 | 0.000 | up | HMDB0004679 |
| Glycoursodeoxycholic acid | 180vs150 | 0.547 | 1.917 | 0.044 | down | HMDB0000708；LMST05030016 |
| Taurochenodeoxycholic acid | 90vs60 | 0.585 | 1.348 | 0.027 | down | HMDB0000951；LMST05040005 |
|  | 120vs90 | 0.409 | 1.216 | 0.006 | down | HMDB0000951；LMST05040005 |
|  | 150vs120 | 2.748 | 1.432 | 0.001 | up | HMDB0000951；LMST05040005 |
| Hexanoylcarnitine | 120vs90 | 18.542 | 1.947 | 0.000 | up | HMDB0000705；LMFA07070070 |
|  | 150vs120 | 0.050 | 1.792 | 0.001 | down | HMDB0000705；LMFA07070070 |
|  | 180vs150 | 2.169 | 1.049 | 0.003 | up | HMDB0000705；LMFA07070070 |
| Heptadecanoic Acid | 120vs90 | 0.638 | 1.042 | 0.012 | down | HMDB0006497；LMFA01010017 |
|  | 150vs120 | 1.859 | 1.334 | 0.004 | up | HMDB0006497；LMFA01010017 |
| LPE 20:5 | 120vs90 | 0.272 | 1.511 | 0.000 | down | LMGP02050053 |
| Deoxycholic acid | 150vs120 | 2.460 | 1.308 | 0.006 | up | HMDB0000626；LMST04010040 |
|  | 180vs150 | 0.472 | 1.873 | 0.023 | down | HMDB0000626；LMST04010040 |
| 9,10-Dihome | 150vs120 | 1.758 | 1.400 | 0.014 | up | HMDB0031679；LMFA02000229 |
| Palmitoylcarnitine | 120vs90 | 3.993 | 1.259 | 0.018 | up | HMDB0000222；LMFA07070004 |
|  | 150vs120 | 0.132 | 1.730 | 0.001 | down | HMDB0000222；LMFA07070004 |
| PC (15:0/15:0) | 150vs120 | 0.368 | 1.065 | 0.045 | down | LMGP01010530 |
| Cortisol | 120vs90 | 0.455 | 1.668 | 0.013 | down | HMDB0000063；LMST02030001 |
|  | 150vs120 | 2.373 | 1.691 | 0.009 | up | HMDB0000063；LMST02030001 |
| Lauric acid ethyl ester | 150vs120 | 1.518 | 1.113 | 0.023 | up | HMDB0033788 |
| 9-KODE | 150vs120 | 1.977 | 1.349 | 0.004 | up | HMDB0004669；LMFA02000274 |
| cis-2-Decenoic acid | 120vs90 | 0.456 | 1.305 | 0.003 | down | HMDB0010726 |
| LPC 18:3 | 180vs150 | 0.007 | 3.813 | 0.000 | down | LMGP01050128 |
| PC (18:1/20:4) | 150vs120 | 0.200 | 1.294 | 0.013 | down | LMGP01010905 |
| 13-HPODE | 150vs120 | 1.994 | 1.577 | 0.001 | up | HMDB0003871；LMFA02000034 |
| LPS 20:5 | 120vs90 | 0.428 | 1.067 | 0.000 | down | LMGP03050027 |
| Adrenosterone | 120vs90 | 0.594 | 1.390 | 0.026 | down | HMDB0006772 |
|  | 150vs120 | 1.634 | 1.236 | 0.029 | up | HMDB0006772 |
| LPS 18:2 | 120vs90 | 0.581 | 1.269 | 0.014 | down | LMGP03050011 |
| LPS 16:1 | 90vs60 | 2.515 | 2.468 | 0.008 | up | LMGP03050010 |
|  | 120vs90 | 0.493 | 1.135 | 0.040 | down | LMGP03050010 |
| Oleic Acid | 120vs90 | 0.308 | 1.173 | 0.006 | down | HMDB0000207；LMFA01030002 |
| Trigoneoside Xb | 120vs90 | 0.432 | 1.596 | 0.012 | down | HMDB0036484 |
|  | 150vs120 | 2.147 | 1.481 | 0.016 | up | HMDB0036484 |
| Taurolithocholic acid 3-sulfate | 90vs60 | 0.459 | 2.204 | 0.003 | down | HMDB0002580 |
| Mevalonic acid | 90vs60 | 1.540 | 2.075 | 0.006 | up | HMDB0000227；LMFA01050352 |
|  | 120vs90 | 0.606 | 1.484 | 0.001 | down | HMDB0000227；LMFA01050352 |
| Ergocalciferol | 120vs90 | 0.497 | 1.279 | 0.019 | down | HMDB0000900；LMST03010001 |
| LPE 14:1 | 120vs90 | 0.310 | 1.411 | 0.016 | down | LMGP02050034 |
| Perillartine | 120vs90 | 0.554 | 1.100 | 0.016 | down | HMDB0035652 |
| Docosapentaenoic acid | 150vs120 | 2.394 | 1.432 | 0.002 | up | HMDB0001976；LMFA04000049 |
| LPE 22:6 | 90vs60 | 0.599 | 1.482 | 0.006 | down | LMGP02050060 |
|  | 150vs120 | 2.591 | 1.628 | 0.001 | up | LMGP02050060 |
| Thromboxane B1 | 90vs60 | 1.651 | 1.258 | 0.034 | up | LMFA03030008 |
|  | 150vs120 | 1.928 | 1.192 | 0.019 | up | LMFA03030008 |
| Ursodeoxycholic acid | 90vs60 | 0.286 | 2.361 | 0.003 | down | HMDB0000946；LMST04010033 |
|  | 150vs120 | 2.805 | 1.568 | 0.000 | up | HMDB0000946；LMST04010033 |
| Hydrocortisone acetate | 120vs90 | 0.321 | 1.718 | 0.001 | down | HMDB0000063 |
|  | 150vs120 | 2.448 | 1.334 | 0.005 | up | HMDB0000063 |
| 16-Hydroxyhexadecanoic acid | 120vs90 | 0.245 | 1.321 | 0.008 | down | HMDB0006294 |
|  | 150vs120 | 3.839 | 1.501 | 0.000 | up | HMDB0006294 |
| Hexadecanamide | 90vs60 | 1.607 | 1.551 | 0.043 | up | HMDB0012273；LMFA08010009 |
| Dehydrocholic acid | 90vs60 | 0.568 | 1.776 | 0.000 | down | LMST04010106 |
| 20-Carboxy-Leukotriene B4 | 90vs60 | 2.411 | 1.697 | 0.004 | up | HMDB0006059；LMFA03020016 |
|  | 120vs90 | 0.303 | 1.594 | 0.001 | down | HMDB0006059；LMFA03020016 |
|  | 150vs120 | 3.438 | 1.632 | 0.001 | up | HMDB0006059；LMFA03020016 |
| N-Oleoyl Dopamine | 180vs150 | 0.464 | 1.472 | 0.045 | down | LMFA08020140 |
| Avocadyne 1-acetate | 180vs150 | 1.794 | 1.835 | 0.009 | up | HMDB0031048；LMFA05000644 |
| Xanthohumol | 120vs90 | 0.502 | 1.353 | 0.003 | down | HMDB0037479；LMPK12120294 |
| Tetradecanedioic acid | 120vs90 | 0.590 | 1.204 | 0.001 | down | HMDB0000872；LMFA01170018 |
|  | 150vs120 | 1.862 | 1.324 | 0.001 | up | HMDB0000872；LMFA01170018 |
| PC (18:0/18:0) | 180vs150 | 0.469 | 2.903 | 0.000 | down | LMGP01010006 |
| SM (d18:0/16:0) | 90vs60 | 2.877 | 1.363 | 0.013 | up | LMSP03010004 |
| LPS 22:6 | 90vs60 | 0.639 | 1.234 | 0.049 | down | LMGP03050013 |
|  | 150vs120 | 2.764 | 1.731 | 0.000 | up | LMGP03050013 |
| Jasmonic acid | 90vs60 | 1.611 | 1.224 | 0.008 | up | HMDB0032797；LMFA02020001 |
|  | 120vs90 | 0.364 | 1.712 | 0.000 | down | HMDB0032797；LMFA02020001 |
| Propionylcarnitine | 120vs90 | 3.743 | 1.305 | 0.004 | up | HMDB0000824；LMFA07070105 |
| Tetrahydrocorticosterone | 150vs120 | 0.076 | 1.635 | 0.013 | down | HMDB0000268；LMST02030100 |
| Methenolone | 120vs90 | 0.469 | 1.140 | 0.020 | down | HMDB0041928；LMST02020028 |
|  | 180vs150 | 2.554 | 2.403 | 0.000 | up | HMDB0041928；LMST02020028 |
| LPG 18:3 | 90vs60 | 0.440 | 2.215 | 0.003 | down | LMGP04050032 |
| LPC 22:6 | 150vs120 | 2.298 | 1.425 | 0.001 | up | LMGP01050056 |
| LPA 12:0 | 120vs90 | 5.026 | 1.272 | 0.014 | up | LMGP10050015 |
|  | 150vs120 | 0.095 | 1.825 | 0.001 | down | LMGP10050015 |
| 4-Oxoretinol | 150vs120 | 1.729 | 1.655 | 0.000 | up | HMDB0012329 |
| D-Erythro-sphingosine 1-phosphate | 120vs90 | 9.280 | 1.817 | 0.000 | up | HMDB0000277 |
|  | 150vs120 | 0.090 | 2.031 | 0.000 | down | HMDB0000277 |
| Fasciculic acid C | 150vs120 | 1.671 | 1.256 | 0.036 | up | HMDB0036440；LMST01010390 |
| Cynaropicrin | 120vs90 | 0.332 | 1.747 | 0.010 | down | HMDB0036423 |
|  | 150vs120 | 2.316 | 1.569 | 0.012 | up | HMDB0036423 |
| LPI 22:4 | 150vs120 | 4.251 | 1.872 | 0.000 | up | LMGP06050013 |
| Cholic acid | 180vs150 | 0.282 | 2.400 | 0.010 | down | HMDB0000619；LMST04010001 |
| LPC 17:2 | 180vs150 | 0.321 | 2.530 | 0.000 | down | LMGP01050127 |
| Soyasaponin I | 150vs120 | 1.786 | 1.550 | 0.001 | up | HMDB0034649 |
| Palmitic Acid | 120vs90 | 0.581 | 1.324 | 0.017 | down | HMDB0000220；LMFA01010001 |
| Prostaglandin E1 | 120vs90 | 0.603 | 1.421 | 0.007 | down | HMDB0001442；LMFA03010134 |
| Geranylgeranyl pyrophosphate | 120vs90 | 0.279 | 1.318 | 0.001 | down | HMDB0004486 |
|  | 150vs120 | 3.251 | 1.223 | 0.001 | up | HMDB0004486 |
| Glycerol 1-hexadecanoate | 90vs60 | 0.430 | 1.582 | 0.000 | down | HMDB0011564 |
|  | 150vs120 | 3.129 | 1.186 | 0.001 | up | HMDB0011564 |
|  | 180vs150 | 2.647 | 1.964 | 0.003 | up | HMDB0011564 |
| Pentadecanoic Acid | 150vs120 | 3.672 | 1.449 | 0.001 | up | HMDB0000826；LMFA01010015 |
| PC (4:0/4:0) | 150vs120 | 0.462 | 1.423 | 0.015 | down | LMGP01011222 |
| 3b,7b-Dihydroxy-5-androsten-17-one | 120vs90 | 0.448 | 1.187 | 0.020 | down | HMDB0004624 |
| Hydrocortisone | 120vs90 | 0.645 | 1.205 | 0.037 | down | HMDB0000063 |
|  | 150vs120 | 1.643 | 1.249 | 0.018 | up | HMDB0000063 |
| 1,4-dihydroxyheptadec-16-en-2-yl acetate | 150vs120 | 2.385 | 1.458 | 0.000 | up | LMFA05000641 |
| Chrysin | 180vs150 | 0.557 | 2.376 | 0.016 | down | HMDB0036619；LMPK12110189 |
| LPI 16:0 | 120vs90 | 0.526 | 1.170 | 0.047 | down | LMGP06050002 |
| LPE 22:5 | 150vs120 | 2.751 | 1.419 | 0.002 | up | LMGP02050070 |
| 7-Ketocholesterol | 90vs60 | 3.844 | 2.142 | 0.005 | up | HMDB0000501 |
| Decanoylcarnitine | 120vs90 | 5.557 | 1.379 | 0.039 | up | HMDB0000651；LMFA07070059 |
|  | 150vs120 | 0.124 | 1.695 | 0.014 | down | HMDB0000651；LMFA07070059 |
| Acetyl-L-carnitine | 120vs90 | 3.085 | 2.023 | 0.000 | up | HMDB0000201；LMFA07070050 |
| O-Arachidonoyl ethanolamine | 90vs60 | 0.274 | 2.252 | 0.000 | down | HMDB0013655 |
|  | 180vs150 | 4.033 | 3.064 | 0.000 | up | HMDB0013655 |
| Thromboxane B2 | 120vs90 | 0.591 | 1.278 | 0.040 | down | HMDB0003252；LMFA03030002 |
| Docosahexaenoic acid | 90vs60 | 0.538 | 1.773 | 0.007 | down | HMDB0002183；LMFA01031176 |
|  | 150vs120 | 2.207 | 1.203 | 0.019 | up | HMDB0002183；LMFA01031176 |
| Prostaglandin A1 | 120vs90 | 0.284 | 1.711 | 0.000 | down | HMDB0002656；LMFA03010005 |
|  | 150vs120 | 2.912 | 1.400 | 0.002 | up | HMDB0002656；LMFA03010005 |
| Citraconic acid | 150vs120 | 0.361 | 1.179 | 0.026 | down | HMDB0000634；LMFA01170099 |
| Genistein | 90vs60 | 0.623 | 1.028 | 0.019 | down | HMDB0003217；LMPK12050218 |
|  | 120vs90 | 0.326 | 1.394 | 0.000 | down | HMDB0003217；LMPK12050218 |
|  | 150vs120 | 6.556 | 2.325 | 0.000 | up | HMDB0003217；LMPK12050218 |
| 5-OxoETE | 150vs120 | 3.025 | 1.182 | 0.000 | up | HMDB0010217 |
| Arachidonic acid | 120vs90 | 0.661 | 1.180 | 0.032 | down | HMDB0001043；LMFA01030001 |
| Lithocholic Acid | 120vs90 | 0.301 | 1.661 | 0.008 | down | HMDB0000761；LMST04010003 |
|  | 150vs120 | 2.807 | 1.521 | 0.015 | up | HMDB0000761；LMST04010003 |
| 5beta-Androstane-3,17-dione | 120vs90 | 0.231 | 1.459 | 0.005 | down | HMDB0003769；LMST02020058 |
|  | 150vs120 | 3.553 | 1.430 | 0.000 | up | HMDB0003769；LMST02020058 |
